# Malignant transformation in a defined genetic background: proteome changes displayed by 2D-PAGE

**DOI:** 10.1186/1476-4598-9-254

**Published:** 2010-09-22

**Authors:** Stephanie M Pütz, Fotini Vogiatzi, Thorsten Stiewe, Albert Sickmann

**Affiliations:** 1 Rudolf Virchow Center, DFG Research Center for Experimental Biomedicine, University of Würzburg, (Protein Mass Spectrometry and Functional Proteomics), D-97078 Würzburg, Germany; 2 Institute of Medical Radiation and Cell Research (MSZ), University of Würzburg, D-97078 Würzburg, Germany; 3 Molecular Oncology, Philipps-University Marburg, D-35032 Marburg, Germany; 4 Leibniz-Institut für Analytische Wissenschaften - ISAS - e.V., D-44227 Dortmund, Germany

## Abstract

**Background:**

Cancer arises from normal cells through the stepwise accumulation of genetic alterations. Cancer development can be studied by direct genetic manipulation within experimental models of tumorigenesis. Thereby, confusion by the genetic heterogeneity of patients can be circumvented. Moreover, identification of the critical changes that convert a pre-malignant cell into a metastatic, therapy resistant tumor cell, however, is one necessary step to develop effective and selective anti-cancer drugs. Thus, for the current study a cell culture model for malignant transformation was used: Primary human fibroblasts of the BJ strain were sequentially transduced with retroviral vectors encoding the genes for hTERT (cell line BJ-T), simian virus 40 early region (SV40 ER, cell line BJ-TE) and H-Ras V12 (cell line BJ-TER).

**Results:**

The stepwise malignant transformation of human fibroblasts was analyzed on the protein level by differential proteome analysis. We observed 39 regulated protein spots and therein identified 67 different proteins. The strongest change of spot patterns was detected due to integration of SV40 ER. Among the proteins being significantly regulated during the malignant transformation process well known proliferating cell nuclear antigen (PCNA) as well as the chaperones mitochondrial heat shock protein 75 kDa (TRAP-1) and heat shock protein HSP90 were identified. Moreover, we find out, that TRAP-1 is already up-regulated by means of SV40 ER expression instead of H-Ras V12. Furthermore Peroxiredoxin-6 (PRDX6), Annexin A2 (p36), Plasminogen activator inhibitor 2 (PAI-2) and Keratin type II cytoskeletal 7 (CK-7) were identified to be regulated. For some protein candidates we confirmed our 2D-PAGE results by Western Blot.

**Conclusion:**

These findings give further hints for intriguing interactions between the p16-RB pathway, the mitochondrial chaperone network and the cytoskeleton. In summary, using a cell culture model for malignant transformation analyzed with 2D-PAGE, proteome and cellular changes can be related to defined steps of tumorigenesis.

## Introduction

The stepwise accumulation of genetic alterations in normal cells is estimated to be a major cause of cancer. One approach to study cancer development is direct genetic manipulation of primary cells to generate experimental models of tumorigenesis. Traditionally, murine cells or transgenic mouse models have been the primary targets of investigation and have provided crucial insights into the molecular mechanisms underlying cancer development [1].

However, cancer biology of murine and human tissues clearly differs [2]. For example, primary human cells cannot be transformed with most combinations of oncogenes that readily induce transformation of primary rodent cells. In addition, prolonged culture of mouse embryonic fibroblasts (MEFs) results in their spontaneous immortalization, whereas comparable treatment of human fibroblasts leads to replicative senescence [2]. This phenomenon can be partially attributed to telomere biology: unlike murine embryonic fibroblasts, primary human fibroblasts have relatively short telomeres and lack detectable telomerase activity.

The majority of human tumor cells are telomerase-positive and expression of the catalytic subunit of the telomerase holoenzyme (hTERT) is sufficient to immortalize a variety of human primary cell types [3,4]. For example, SV40 LT transfected human fibroblasts have an extended lifetime but undergo crisis that can be rescued by expression of hTERT. Consistently, Hahn and co-workers succeeded to transform human primary fibroblasts and epithelial cells with the classical oncogenes H-Ras V12 and the transforming early region of simian virus 40 (SV40 ER) following initial expression of hTERT in primary cells [5]. Subsequent work from many laboratories has confirmed the results and used the same combination of introduced genes to convert a wide variety of primary human cells to tumor cells, including human mammary epithelial, airway epithelial, glial, endothelial and mesothelial cells [6-10].

Whereas induction of hTERT and activating mutations of H-Ras are frequently observed in human tumors, the small DNA tumor virus SV40 does not appear to be a common cause of human cancer. However, a dissection of the signaling pathways affected by SV40 ER has revealed striking similarities between SV40 ER function and alterations seen in human tumors [11]. The SV40 ER produces two major gene products, the large tumor antigen (LT) and the small tumor antigen (ST). LT is known to bind to and modulate the action of many host cell proteins, however, its role in the transformation of human cells appears to lie solely in the inactivation of the two major tumor suppressors, p53 and RB [11]. Consistently, specific siRNAs directed against RB and p53 can replace the requirement for LT in these experiments [12]. In contrast, ST, which inactivates the Serine/threonine-protein phosphatase (PP2A) via binding to the A and C subunits, exerts its oncogenic potential, at least partially, by preventing dephosphorylation of c-Myc, resulting in c-Myc stabilization [13-15]. Moreover, a stable c-MycT58A mutant that cannot be dephosphorylated by PP2A replaces SV40 ST in human cell transformation and tumorigenesis assays. Considering that c-Myc is one of the first identified oncogenes, it can be concluded that the transforming early region of SV40 appears to target the same cellular signaling pathways that are frequently affected during human tumorigenesis. In turn this renders the model of human cell transformation by the combination of the defined genetic elements hTERT, SV40 ER and H-Ras V12 an attractive model system to analyze changes during human cell transformation in molecular terms.

**Figure 1:**
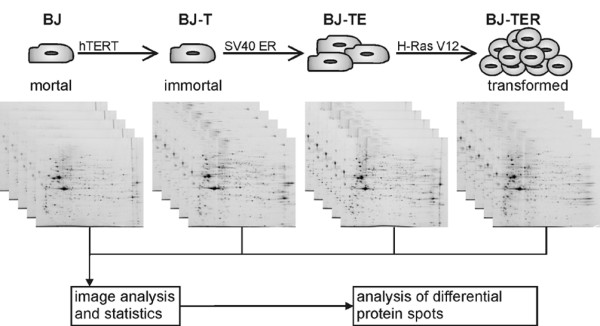
**Cell culture model for malignant transformation and experimental workflow**. The primary human fibroblast cell line BJ was sequentially transduced with retroviruses encoding hTERT, SV40 ER and H-Ras V12 to generate the fully transformed cell line BJ-TER showing the characteristics of cancer cells. Five independent biological replicates were analyzed for BJ, BJ-T, BJ-TE and BJ-TER.

Progress in genomic and proteomic technologies (e.g. sensitive and fast MS and MS/MS analysis of proteins and peptides) promise to identify the characteristic signatures of specific cancer subtypes, to improve the classification of tumor types, and to identify prognostically relevant markers. However, these studies are always confounded by the genetic heterogeneity of patients. Thus, understanding the development of the transformed phenotype therefore requires analysis of the various stages of tumorigenesis in a defined genetic background. Whole-genome-scale technology then allows the identification of the molecular changes during each transition. As a first approach, we analyzed the stepwise transformation of human fibroblasts by a proteomic technique. Therefore, human primary fibroblasts of the BJ strain, that were sequentially transduced with retroviral vectors encoding the genes for hTERT (BJ-T), SV40 ER (BJ-TE) and H-Ras V12 (BJ-TER), were used (Figure 1) [16]. Whereas the mortal BJ fibroblasts enter replicative senescence after prolonged culture, BJ-T cells prevent senescence due to expression of active telomerase. BJ-TE cells have disrupted RB- and p53-regulated checkpoints but remain anchorage-dependent and non-tumorigenic. In contrast, BJ-TER cells represent a fully transformed phenotype including tumorigenicity in vivo, as previously shown [5]. For visualizing different proteomes, two-dimensional polyacrylamide gelelectrophorese (2D-PAGE) is the method of choice [17-19]. Therefore, this technique was used to get an overview of the proteome of the described cell lines that represent defined stages of the malignant transformation process. Our results give new insights in the proteome changes following the stepwise transformation of human cells. We present the characterization of 39 distinct differentially regulated protein spots which contain 67 proteins including PCNA, Peroxiredoxin-6 (PRDX6), Plasminogen activator inhibitor 2 (PAI-2), the cytoskeletal protein cytokeratin-7 (CK-7) and the cytoskeletal associated protein Annexin A2 (p36) as well as the heat shock proteins TRAP-1, HSP90-alpha and HSP90-beta.

## Materials and methods

### Malignant Transformation - Generation of cell lines

The cell lines BJ-T, BJ-TE and BJ-TER (Figure 1) were generated by sequential infection of the human diploid fibroblast strain BJ (ATCC CRL-2522) with retroviruses that were produced by transfection of the amphotropic packaging cell line PT67 (Becton Dickinson, Heidelberg, Germany) with the plasmids pWZL-blast3-hTERT, pZIP-SV776-1 or pLPC-HrasV12 [5,20,21]. Transduced cells were selected with 1 μg/ml blasticidin (Merck, Darmstadt, Germany), 400 μg/ml G418 (PAA, Pasching, Austria), or 0.75 μg/ml puromycin (Becton Dickinson), respectively. All cell lines were maintained in Dulbecco's Modified Eagle's Medium (Sigma, München, Germany) supplemented with 10% fetal bovine serum (Sigma), 1% penicillin G/streptomycin (Invitrogen, Karlsruhe, Germany) and 0.4% Amphotericin (Sigma). Media were changed twice a week and cells were grown in a humidified atmosphere with 5% CO_2_.

### Sample preparation

Confluent cell plates with a diameter of 12 cm were washed twice with PBS (Sigma). Next, cells were scraped from the plate in 2 mL PBS and centrifuged for 10 min at 400 ×g and 4°C. The resulting pellet was lysed with 7 M urea (AppliChem, Darmstadt), 2 M thiourea (Merck), 2% CHAPS (Merck) supplemented with 1 tablet protease inhibitor Complete Mini (Roche) per 10 mL buffer. Subsequently, samples were sonicated six times in an ultrasonic bath for 10 seconds with cooling intervals of one minute. Afterwards samples were centrifuged at 16000 ×g for 10 min. The supernatant was stored at -80°C. The protein amount was determined by Amido Black assay [22].

### 2D-PAGE

Immediately before 2D-PAGE separation, 100 μg of protein samples were diluted with IEF buffer, containing 7 M urea, 2 M thiourea, 2% CHAPS, 5% 2,2'-Dithiodiethanol (Sigma) and 2% IPG buffer (GE Healthcare, Munich, Germany) to a final volume of 100 μL. That way prepared samples were used for 2D-PAGE as described elsewhere [17]. For the first dimension 24 cm IPG strips (3-10 NL) (GE Healthcare) and sample cups were used. Isoelectric focusing was performed for a total of 50 kVh (hold 150 V for 2 h, hold 300 V for 2 h, ramp to 500 V in 2 h, ramp to 1000 V in 3 h, ramp to 4000 V in 3 h, hold at 6000 V for 7 h) using an Ettan IPGphor (GE Healthcare). After IEF, IPG strips were equilibrated in two steps each 20 min in 6 M urea, 30% glycine, 2% SDS, 50 mM Tris HCl, pH 8.8 supplemented with 130 mM DTT and 280 mM iodoacetamide respectively. Subsequently, IPG strips were placed on top of 10% polyacrylamide gels and Mark 12 (Invitrogen) was used as molecular weight marker for SDS-PAGE. The second dimension was carried out using the Ettan DALTSix (GE Healthcare). At the beginning 5 W/gel were applied for 30 min and afterwards 17 W/gel or max. 100 W until the running front reached the end of the glass plate. Gels were fluorescence stained with RuPBS as described elsewhere [23,24]. Scanned gels were compared and analyzed with the image analysis software PDQuest Advanced 8.0.1 (Bio-Rad, Munich, Germany) with BJ cells taken as control. The intensities of the gels were normalized and all protein spots exhibiting a minimum regulation by a factor of two were marked for further manual evaluation. Differential protein spots were excised, washed and digested [25].

### Mass spectrometry and data analysis

Proteins were identified by nano-LC-MS/MS analysis. The analyses were conducted with either an LTQ XL using Xcalibur 2.0.5 and Bioworks 3.3.1 (Thermo-Scientific, Dreieich, Germany), a QStarElite in combination with AnalystQS 2.0 (Applied Biosystems, Darmstadt, Germany) or a Qtrap4000 in conjunction with Analyst 1.4.2 (Applied Biosystems). Samples were preconcentrated using a Synergi Hydro-RP C18 trapping column (100 μm ID, 2 cm length, 80 Å pore size, 4 μm particle size; Phenomenex, Aschaffenburg, Germany) and afterwards separated on a Synergi Hydro-RP C18 main column (75 μm ID, 150 mm length, 80 Å pore size, 2 μm particle size; Phenomenex) using a linear binary gradient (solvent A: 0.1% formic acid in water; solvent B: 0.1% formic acid, 84% acetonitril) at a flow rate of 270 nL/min. Full MS scans from 300 to 1500 or 2000 m/z respectively were acquired, and the three to five most intensive peptide ions were subjected to further fragmentation, depending on the mass spectrometer. Duplicate detection of a single m/z within 30 s led to dynamic exclusion.

LTQ raw data-files were converted into mgf-files using the supplied version of LCQ-DTA.EXE as plug-in to Mascot Daemon with the following parameters: (a) minimum mass: 400, (b) maximum mass 3000, (c) grouping tolerance 1.4, (d) min. scans/group: 1, (e) intermediate scans: 1, (f) precursor charge: auto. QStarElite and Qtrap4000 wiff-files were converted into mgf-files using the respective mascot.dll with the parameters: (a) precursor mass tolerance for grouping 0.05 (QStarElite) 0.2 (Qtrap), (b) max. number cycles between groups 4 (QStarElite) 1 (Qtrap), (c) min. number cycles per group 1, (d) Remove peaks if intensity < 0.05 (QStarElite) < 0.1 (Qtrap) % of maximum, (e) centroid all MS/MS data, (f) reject spectra if less than 10 peaks. Protein identification was performed using Mascot™, Version 2.2.0 (Matrix Science, London, UK) and the SwissProt database (02-03-2006) with 208005 entries. Mascot parameters were choosen as follows: protease: trypsin, fixed modifications: carbamidomethyl (C), variable modifications: oxidation (M), taxonomy: Homo sapiens (13538 entries), missed cleavages: 2, peptide and MS/MS tolerance: ± 0.2 (QStarElite) ± 0.4 (Qtrap) ± 1.5 (LTQ), significance threshold: p < 0.05. For spots not satisfactorily identified with the SwissProt database an additional search was conducted against human IPI (version v3.26, 67665 entries) using Mascot™, Version 2.1 (Matrix Science) with the same parameters used for SwissProt. Significant MS/MS peptide identifications were verified manually.

### Data interpretation

Hierarchical clustering and visualization of the intensities of regulated protein spots according to cell line and regulation pattern was accomplished as described elsewhere [26]. Subcellular localization of identified proteins was extracted from the UniProt database http://www.uniprot.org/ in December 2009. At the same time functional annotation of the identified proteins was allocated using the Go Term Mapper website http://go.princeton.edu/cgi-bin/GOTermMapper and the human GO Slim categories. Identified proteins were grouped into the categories for biological process.

### Western Blot

Cells pellets, previously flash frozen in liquid nitrogen and stored at -80°C, were lysed in NP40 buffer (50 mM Tris-HCl ph 8.0, 150 mM NaCl, 5 mM EDTA ph 8.0, 2% NP40) supplemented with protease inhibitor cocktail (Roche). 30 μg of soluble protein was separated on 4-12% NuPAGE Bis-Tris gels (Invitrogen) and transferred to ECL nitrocellulose membranes by wet blotting. Membranes were blocked with 10% non fat dry milk in TBST (5 mM Tris, 15 mM NaCl, pH 7.6, 0.1% Tween20). Primary antibodies were diluted in TBST with 5% non fat dry milk at the following concentrations: PCNA (sc-56, Santa Cruz, 1 μg/ml), PRDX6 (ab92322, Abcam), HSP90-alpha (AB3466, Millipore, 2 μg/ml), HSP90-beta (AB3468, Millipore, 2 μg/ml), PAI2 (sc-25745, Santa Cruz, 1 μg/ml), and β-Actin (ab6276, Abcam, 1 μg/ml). Detection was performed with horseradish peroxidase or Alexa Fluor 680 linked secondary antibodies using enhanced chemiluminescence (Thermo Fisher) or infrared imaging (Odyssey, LI-COR).

## Results

**Figure 2:**
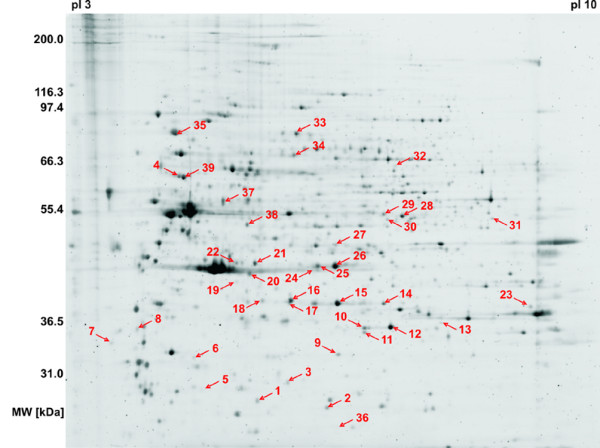
**Proteome visualization of the malignant transformation model cell lines by 2D-PAGE**. A representative 2D-gel from the cell line BJ stained with RuBPS is shown. Regulated protein spots are indicated with arrows and numbers. The degree of regulation for these protein spots is given in Figure 3, while identified proteins are listed in Table 2.

The cell culture model for malignant transformation consisting of the four cell lines BJ, BJ-T, BJ-TE and BJ-TER was analyzed by a differential 2D-PAGE approach. To compare the protein spot patterns of the different cell lines, a minimum of five cell plates was processed to obtain five independent biological replicates for each cell line (gels shown in Additional file 1). Analysis and subsequent manual validation of the PDQuest output resulted in 39 differentially regulated protein spots among the four cell lines. Figure 2 shows a representative 2D-PAGE gel from cell line BJ and the protein spots which are regulated among the four cell lines.

**Figure 3:**
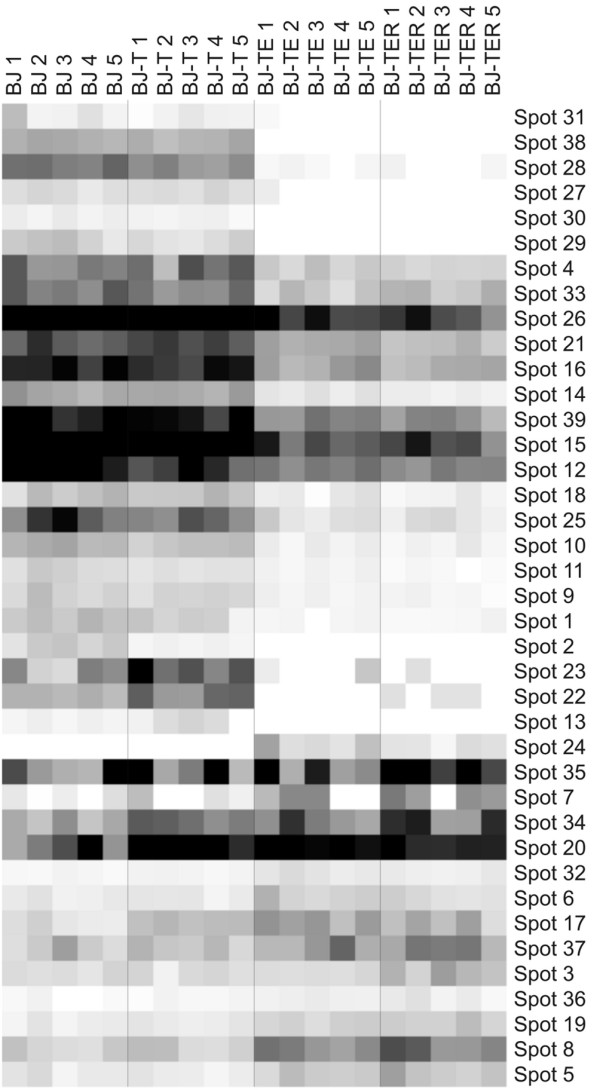
**Hierarchical clustering of intensities of regulated protein spots according to cell line and regulation pattern.** The five 2D-gel replicates from each cell line are numbered; each column represents one gel; each row represents a protein spot. The spot intensities are indicated in grayscale (white = no intensity; black = highest intensity).

To determine which protein spots were regulated by which genetic alteration, the normalized intensities of each regulated protein spot were used for hierarchical clustering. Protein spots were sorted according to their regulation pattern using the software Cluster [26] and results are shown in Figure 3. It is evident that many protein spots show their strongest regulation between the cell lines BJ-T and BJ-TE caused by the insertion of SV40 ER. During the malignant transformation from BJ (via BJ-T and BJ-TE) to BJ-TER the protein spots become continuously up- or down-regulated except a few spots. As an example spot 22 shows increased peak intensity in stage BJ-T compared to BJ and is absent in BJ-TE, but up-regulated again due to the H-Ras transition. Therefore, it may be concluded that the majority of the regulated proteins during malignant transformation is either continuously up- or down-regulated. Moreover, for most protein spots the in-group variability is low, indicating good reproducibility. Beside the visualization of the hierarchical clustering, the number of spots up-or down-regulated within the transitions is summarized in Table 1. In addition, spots that were not detectable before or after a transition are indicated.

**Table 1:** Spots regulated within the transitions and the number of identified proteins

transition	up-regulated spots	sum of up- regulated spots	down-regulated spots	sum of down- regulated spots	sum of identified proteins
**BJ → BJ-T** **(hTERT)**	13, 17, 20, 23	4	2, 31	2	7

**BJ-T → BJ-TE** **(SV40)**	5, 6, 7, 8, 24*, 32	6	1, 2*, 4, 9, 10, 11, 13*, 14, 16, 18, 21, 22*, 23, 25, 26, 28, 29*, 30*, 31, 33, 38*	21	39

**BJ-TE → BJ-TER** **(RAS)**	22*, 35	2	27*, 31*	2	8

**BJ → BJ-TE** **(hTERT + SV40)**	5, 6, 7, 8, 17, 19, 24*, 32	8	1, 2*, 4, 9, 10, 11, 12, 13*, 14, 15, 16, 18, 22*, 23, 25, 26, 28, 29*, 30*, 31, 33, 38*, 39	23	48

**BJ-T → BJ-TER** **(SV40 + RAS)**	3, 5, 7, 8, 19, 24*, 35, 36	8	1, 2*, 4, 9, 10, 11, 13*, 14, 16, 18, 21, 22, 23, 25, 26, 27*, 28, 29*, 30*, 31*, 38*, 39	22	47

**BJ → BJ-TER** **(all)**	3, 5, 7, 8, 17, 19, 24*, 32, 34, 35, 36, 37	12	1, 2*, 4, 9, 10, 11, 12, 13*, 14, 15, 16, 18, 21, 22, 23, 25, 26, 27*, 28, 29*, 30*, 31*, 33, 38*, 39	25	63

The numbers of spots up- or down-regulated within the transitions are listed and summed up. The number of identified proteins within regulated spots of a particular transition is presented in the right column. Spots marked with an asterisk are not detectable before or after a transition.

Identification of the proteins in differentially regulated 2D-PAGE spots was accomplished by nano-LC-MS/MS. While 39 protein spots showing significant changes in intensity, 32 could be readily identified, corresponding to an identification rate of 82%. Moreover, for 25 protein spots more than a single protein was identified. Table 2 lists the identified proteins according to the spot number. Regulation of spot 24 could not be determined, because this protein spot is absent in the gels of the cell lines BJ and BJ-T. Therefore the normalized intensities and the error estimated by PDQuest were included in Table 2 instead. For more detailed protein information (e.g. MW, pI) also see the Additional file 1. In total, the analysis of the 39 differential protein spots resulted in the identification of 67 proteins.

**Table 2:** Identified proteins within the protein spots showing at least two-fold up- or down-regulation between the cell lines of the malignant transformation model.

Spot No	SwissProt ID	gene	BJ	T	TE	TER	IPI ID
1	P04792	HSPB1	1,00	0,69	***0,17***	***0,13***	
	Q13242	SFRS9					

2	P30041	PRDX6	1,00	***0,25***	***0,00***	***0,00***	

3	Q06323	PSME1	1,00	0,98	1,03	**2,05**	
	P35232	PHB					

4	Q9UNF0	PACSIN2	1,00	1,10	***0,40***	***0,34***	

5	P43487	RANBP1	1,00	0,79	**2,18**	**2,61**	
	P25788	PSMA3					
	P07686	HEXB					

6	Q15691	MAPRE1	1,00	1,03	**2,46**	1,69	
	Q99426	TBCB					
	P61247	RPS3A					

7	N.D.		1,00	1,99	**5,53**	**5,98**	

8	P12004	PCNA	1,00	1,10	**2,59**	**2,97**	
	P01584	IL1B					

9	N.D.		1,00	0,88	***0,27***	***0,20***	

10	N.D.		1,00	0,76	***0,21***	***0,16***	

11	O00463	TRAF5	1,00	0,71	***0,30***	***0,16***	

12	P13716	ALAD	1,00	0,72	***0,48***	***0,44***	
	O14656	TOR1A					

13	P07355	ANXA2	1,00	**2,58**	***0,00***	***0,00***	

14	P31942	HNRPH3	1,00	0,99	***0,29***	***0,18***	
	P02545	LMNA					

15	N.D.		1,00	0,62	***0,39***	***0,40***	

16	N.D.		1,00	0,95	***0,41***	***0,34***	

17	O60664	M6PRBP1	1,00	**2,30**	**3,26**	**2,40**	
	P06748	NPM1					
	P07195	LDHB					

18	P08670	VIM	1,00	1,03	***0,33***	***0,23***	

19	Q9UJZ1	STOML2	1,00	1,24	**2,46**	**2,81**	
	Q9NWT6	HIF1AN					
	O94905	ERLIN2					

20	P05120	SERPINB2	1,00	**2,18**	1,84	1,50	
	P60709	ACTB					

21	P60709	ACTB	1,00	1,09	0,53	***0,38***	

22	P60709	ACTB	1,00	1,80	***0,00***	***0,39***	
	Q12905	ILF2					
	P60842	EIF4A1					
	O60664	M6PRBP1					

23	N.D.		1,00	**2,14**	***0,42***	***0,36***	

24	Q9Y570	PPME1	0	0	**235347**	**119221**	
	Q6NUK1	SLC25A24					IPI00337494

25	Q9Y570	PPME1	1,00	0,79	***0,20***	***0,16***	
	Q13148	TARDBP					
	P60709	ACTB					

26	P23526	AHCY	1,00	0,85	***0,40***	***0,33***	
	P36507	MAP2K2					
	none	none					IPI00410404
	Q6NUK1	SLC25A24					IPI00337494

27	P06733	ENO1	1,00	1,07	0,57	***0,00***	
	P68104	EEF1A1					

28	P09913	IFIT2	1,00	0,79	***0,06***	***0,08***	
	P00352	ALDH1A1					
	Q15813	TBCE					
	P12268	IMPDH2					
	O43175	PHGDH					
	P11413	G6PD					

29	P00352	ALDH1A1	1,00	0,71	***0,00***	***0,00***	
	O43175	PHGDH					
	Q9UMS4	PRPF19					

30	P11413	G6PD	1,00	0,74	***0,00***	***0,00***	
	P28838	LAP3					

31	N.D.		1,00	***0,49***	***0,23***	***0,00***	

32	Q12931	TRAP1	1,00	1,36	**3,16**	**2,15**	

33	P06396	GSN	1,00	0,88	***0,36***	***0,48***	
	Q96TA1	FAM129B					

34	P20591	MX1	1,00	1,78	1,59	**2,10**	
	P13667	PDIA4					
	P11021	HSPA5					
	P17812	CTPS					

35	P08238	HSP90AB1	1,00	0,99	0,97	**2,07**	
	P07900	HSP90AA1					

36	P30048	PRDX3	1,00	0,94	1,86	**2,07**	
	P62491	RAB11A					
	P27635	RPL10					

37	P10809	HSPD1	1,00	1,10	1,79	**2,09**	
	P68363	K-ALPHA-1					
	P07237	P4HB					
	P60709	ACTB					
	P54578	USP14					
	P48643	CCT5					

38	P08729	KRT7	1,00	0,96	***0,00***	***0,00***	

39	Q969P6	TOP1MT	1,00	0,85	***0,43***	***0,38***	
	P08670	VIM					
	P14625	HSP90B1					

Shown are the corresponding SwissProt ID, gene name and the average regulation of the protein spot normalized to the spot intensity in the BJ cells (bold = up-regulation, bold italic = down-regulation). For detailed information see Additional file 1.

**Figure 4:**
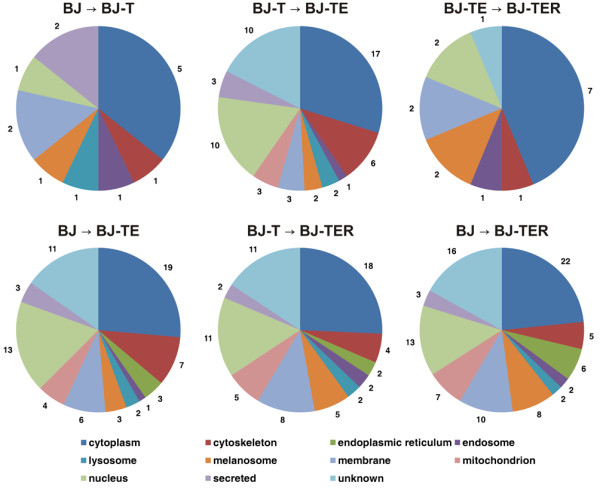
**Subcellular localization.** Subcellular localization of all proteins identified within the protein spots regulated between BJ and BJ-T cells (top left), between BJ-T and BJ-TE (top middle) and BJ-TE and BJ-TER (top right). Additionally the localization-data of multi step transitions are depicted in the lower row. Multiple occurrences per protein were allowed.

**Figure 5:**
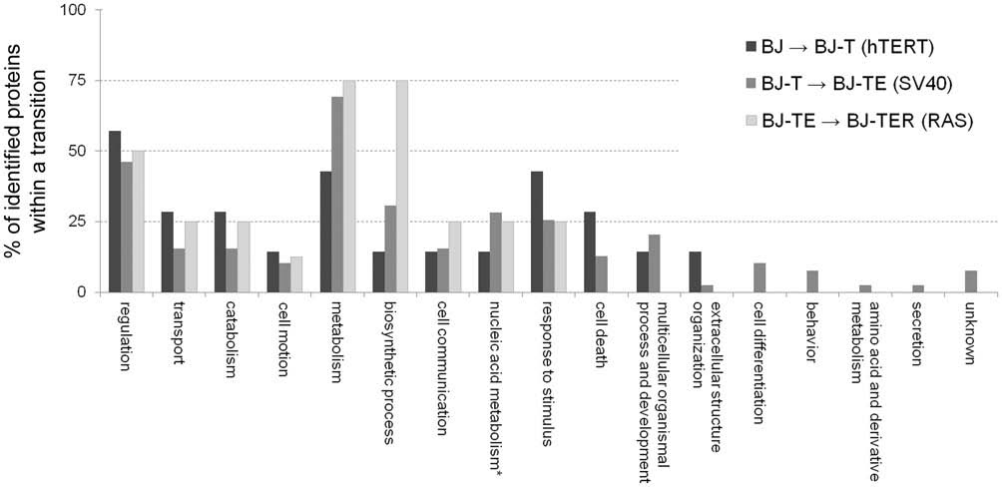
**Function of proteins.** Function of all proteins identified in differential spots. After integration of the genetic elements hTERT (transition from BJ to BJ-T = black), SV40 ER (transition from BJ-T to BJ-TE = dark gray) and H-Ras (transition from BJ-TE to BJ-TER = light gray). Nucleic acid metabolism (marked with an asterisk) comprise also nucleobase, nucleoside and nucleotide metabolism. Multiple occurrences were allowed. The vertical-axis displays the share of all proteins identified within the respective transition.

To investigate a relation between the introduction of a specific genetic alteration and the proteins identified in the thereby regulated spots in terms of function and localization, the protein spots were grouped according to their regulation (as listed in Table 1) and the respective proteins were further analyzed using UniProt database entries for subcellular localization and the Go Term Mapper website and human GO Slim categories for functional annotation. Results are depicted in Figure 4 and Figure 5. Altogether seven proteins were identified within the six differential protein spots between BJ and BJ-T cell line, 39 proteins within the 27 spots between BJ-T and BJ-TE and eight proteins within the four spots that were differentially expressed between BJ-TE and BJ-TER. For the multi-step transitions see Table 1. Regarding the subcellular localization (Figure 4), at least a quarter of the proteins were derived from cytoplasm during all transitions. In contrast, mitochondrial and nuclear proteins were mainly involved in the transition from BJ-T to BJ-TE, whereas proteins localized in the endoplasmic reticulum were found exclusively in the multi-step transitions. Moreover, lysosomal and secreted proteins were not found in the Ras transition. Here it needs to be mentioned that secreted proteins might have been lost during cell culture. Moreover, the separation of membrane proteins by conventional 2D-PAGE is not sufficient and has to be met by alternative separation strategies [27]. Nevertheless, we found some membrane proteins particularly in the multi-step transitions. Analogous to the subcellular localization, functional classification of the identified proteins was carried out and the single-step transitions were shown in Figure 5. Compared to the others, in the hTERT transition fewer amounts of proteins are identified belonging to the GO slim categories metabolism and biosynthetic process. In these categories and cell communication the percentile amount of proteins identified in the H-Ras transition are increased. Proteins involved in cell death, multicellular organismal processes and extracellular structure organization are identified only in the hTERT and SV40 ER transition. Moreover, introduction of SV40 ER also affects proteins involved in secretion, amino acid metabolism, cell differentiation and behavior.

To condense the results found so far, proteins identified within the regulated spots are displayed in Table 3 according to the transitions and the respective features. Interestingly, two proteins, Protein phosphatase methylesterase 1 (PPME1) and Solute carrier family 25 member 24 (SLC25A24), were identified together in the up-regulated spot 24. Within the same transition (SV40 ER) these proteins were also identified in two different down-regulated spots.

**Table 3:** Transition features and the presumably regulated proteins

transition	transition feature	genes presumably up-regulated proteins	genes presumably down-regulated proteins
**BJ → BJ-T** **(hTERT)**	prevention of senescence	ACTB, ANXA2, LDHB, M6PRBP1, NPM1, SERPINB2	PRDX6

**BJ-T → BJ-TE** **(SV40)**	proliferation and prevention of apoptosis	HEXB, IL1B, MAPRE1, PCNA, **PPME1**, PSMA3, RANBP1, RPS3A, **SLC25A24**, TBCB, TRAP1	ACTB, AHCY, ALDH1A1, ANXA2, EIF4A1, FAM129B, G6PD, GSN, HNRPH3, HSPB1, IFIT2, ILF2, IMPDH2, IPI00410404, KRT7, LAP3, LMNA, M6PRBP1, SFRS9, MAP2K2, PACSIN2, PHGDH, **PPME1**, PRDX6, PRPF19, **SLC25A24**, TARDBP, TBCE, TRAF5, VIM

**BJ-TE → BJ-TER** **(RAS)**	prevention of anoikis and growth-factor indepandancy	ACTB, EIF4A1, HSP90AA1, HSP90AB1, ILF2, M6PRBP1	EEF1A1, ENO1

The respective transition features and the genes of proteins identified in the spots up- or down-regulated within the transitions are listed. In one case the protein ID is listed. PPME1 and SLC25A24 were identified both in the up-regulated spot 24 and within the same transition these proteins were also identified in two different down-regulated spots. Hence, they are mentioned within the presumably up- and down-regulated proteins of the SV40 ER transition.

Summarizing the results, we detected 39 differential protein spots within the cell culture model for malignant transformation, identified 67 proteins, and grouped them according to their stage of regulation, localization and function. One major problem of 2D-PAGE is the detection of multiple proteins per spot [17], thus a precise quantification can only be conducted for single-protein spots. Therefore, in a separate table (Table 4), we listed the seven spots containing only a single protein, which was severalfold identified in the replicate gels of the cell line thereby proving the regulation directly. In addition, at least three spectra of different peptides were required for identification to be included into Table 4. The unambiguously regulated proteins are PRDX6, PCNA, p36, PAI-2, TRAP-1 and CK-7. In spot 35 two homologous proteins (HSP90-alpha and HSP90-beta) were identified. The proteins identified in the other 32 protein spots need to be further validated by other methods.

**Table 4:** List of all unequivocally identified proteins

spot no	SwissProt ID	protein	protein name abbreviation	gene	pI	MW	BJ	T	TE	TER	Subcellular localization	selection of biological processes
2	P30041	Peroxiredoxin-6	PRDX6	PRDX6	6,02	24888	1,00	0,25	0,00	0,00	cytoplasm	cell redox homeostasis

8	P12004	Proliferating cell nuclear antigen	PCNA	PCNA	4,57	28750	1,00	1,10	2,59	2,97	nucleus	cell proliferation

13	P07355	Annexin A2	p36	ANXA2	7,56	38449	1,00	2,58	0,00	0,00	secreted	skeletal system development

20	P05120	Plasminogen activator inhibitor 2	PAI-2	SERPINB2	5,46	46566	1,00	2,18	1,84	1,50	cytoplasm, secreted	anti-apoptosis

32	Q12931	Heat shock protein 75 kDa, mitochondrial	TRAP-1	TRAP1	8,3	80060	1,00	1,36	3,16	2,15	mitochondrial	cellular response to oxidative stress and protein folding

35	P08238	Heat shock protein HSP 90-beta	HSP90	HSP90AB1	4,97	83081					cytoplasm	protein folding
							1,00	0,99	0,97	2,07		
	P07900	Heat shock protein HSP 90-alpha	HSP86	HSP90AA1	4,94	84490					cytoplasm	mitochondrial transport and protein refolding

38	P08729	Keratin, type II cytoskeletal 7	CK-7	KRT7	5,23	51418	1,00	0,96	0,00	0,00	cytoplasm, cytoskeleton	cytoskeleton organization

Shown are SwissProt ID, protein name and abbreviation as well as gene, isoelectric point (pI), molecular weight (MW), regulation, subcellular localization, and a selection of the biological processes the proteins are involved.

**Figure 6:**
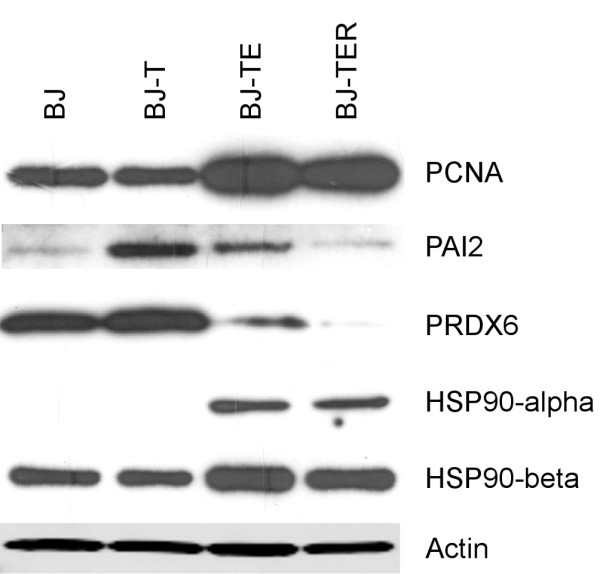
**Western Blots.** Western Blots showing expression of the indicated proteins. (PCNA, PAI2, PRDX6, HSP90-alpha and HSP90-beta) in the four BJ cell lines. β-Actin is shown as a loading control.

Widely known issues of 2D-PAGE are multiple protein identifications per spot and shifting of proteins in the 2D-Gel pattern due to modifications. To verify the expression level for some candidate proteins, Western Blot analyses were conducted (Figure 6). The Western Blots of selected proteins confirm that PCNA is up-regulated due to SV40 ER and PAI2 is most abundant in cell line BJ-T. The homologous proteins HSP90-alpha and HSP90-beta detected by 2D-PAGE within spot 35 can be detected separately with Western Blots. Both HSP90-alpha and HSP90-beta show regulation. Complete HSP90-alpha, HSP90-beta and PRDX6 levels differ from the regulation pattern of the respective 2D-PAGE spots, but show the same direction of regulation considering the full transformation process.

## Discussion

A cell culture model for malignant transformation offers the possibility to study cancer development following specified genetic alterations in front of a defined genetic background. During the last decade, 2D-PAGE has been the standard for separation, visualization and comparison of closely related proteomes. We decided to improve the reliability of results by using a minimum of five cell culture plates from each cell line (Figure 1). Each plate was processed independently giving at least five biologically independent recurrences for each cell line. Only protein spots showing regulation changes independent of the biological variance are relevant changes originating from genetic alterations.

Besides gel to gel variation, the choice of the appropriate protein staining method has major influences on the image analysis with existing software. Commonly, silver or Coomassie staining is used for the detection of proteins in 2D-gels. Silver staining is a very sensitive protein visualization method, but suffers from low reproducibility, because over-staining effects (donut- or crater spots) and potential interference with subsequent MS occurs [28,29]. Although colloidal Coomassie staining offers better compatibility with MS compared to many silver-staining protocols, it is less sensitive [30]. To overcome these limitations we used Ruthenium(II)tris-bathophenanthroline disulfonate (RuBPS) [31]. In comparison to silver staining RuBPS fluorescent staining shows a higher linearity in signal intensity and is more reproducible. Furthermore, RuPBS is more sensitive than Coomassie and offers compatibility with MS [23,24].

In addition to the use of a MS compatible staining method the MS method itself has a major effect on the success of identification. Using nano-LC-MS/MS, we had an identification rate of 82% in this study. A related proteomic approach, however, had a rate of roughly 50% [32], which is most probably due to the use of MALDI-MS. Moreover, it was previously shown that the use of MALDI-MS and LC-ESI-MS/MS in combination achieves the best results [33]. However, superior separation and sensitivity by nano-LC-MS/MS also led to the identification of more than a single protein in several spots - a situation often encountered in 2D-PAGE studies [17]. The utilized mass analyzers are getting more sensitive; thus it is possible to identify also very low abundant proteins in the spots co-migrating in the presence of high abundant proteins. Although, more than 5000 proteins can be resolved by 2D-PAGE approaches simultaneously and ~2000 proteins routinely [17], depending on used gel size and pH gradient. The complexity of complete cell lysates with presumably several hundred thousand protein features [34] can easily surpass the available resolution. Therefore, the detection of more than one protein per spot needs to be expected. In these cases it remains unclear which of the detected proteins is regulated and other methods need to be employed for further validation.

Western Blot analysis is a common method to verify 2D-PAGE results regarding the known restrictions: multiple protein identifications per spot and shifting of proteins in the 2D-gel pattern due to modifications. Our Western Blots support the findings of 2D-PAGE with respect to the full transformation process. However, the 2D-PAGE regulation pattern in some cases differs from the Western Blot results. A possible explanation could be that in 2D-PAGE single protein species are detected whereas in Western Blots the total expression level of a protein including all of its isoforms and modifications is analyzed.

Besides these technical considerations, many changes on the proteome level were detected in the malignant transformation model that are worth to be discussed. BJ-TE cells have disrupted RB- and p53-regulated checkpoints but remain anchorage-dependent and non-tumorigenic. In this step more pathways are altered in a detectable manner than in the other two steps. SV40 LT inactivates RB and p53 due to binding of RB via its N-terminal LXCXE motif and p53 via its bipartite C-terminal binding domain [35]. Unlike SV40 LT the exact contribution of SV40 ST to cellular transformation remains elusive. It is known that SV40 ST binds PP2A, stimulates the phosphorylation of Protein Kinase B (Akt) and affects the c-Myc pathway [13,14].

Besides the transition BJ-T to BJ-TE, the other transitions also significantly affect the proteome as detectable by 2D-PAGE. A thorough study on the H-Ras transformation step was done by Young and coworkers who investigated the proteomic changes associated with expression of H-Ras in a human ovarian epithelial cell line that was immortalized with SV40 LT, ST and the human catalytic subunit of telomerase [32]. They identified 32 proteins that were up- or down-regulated more than 1.5 fold. Moreover, they used a narrower pH range from 4 to 7 resulting in a higher resolution of this part of the proteome. Proteins detected are involved in cellular processes like metabolism, redox balance, calcium signaling, apoptosis and cellular methylation. The unambiguously regulated proteins identified in our current analysis were not detected by their approach. Thus the cell line reflecting the tissue origin possibly has a major not yet understood impact on the proteins regulated by H-Ras.

Cellular localization and function of a list of proteins point to the ongoing molecular changes (Figure 4 and 5). Avoiding cell death and apoptosis is an essential step during malignant transformation. SV40 LT alone bypasses senescence [36,37], but does not fully immortalize human cells, as such modified cells eventually undergo crisis. However, in combination with hTERT immortalization is achieved [11,38]. Thus, finding proteins involved in cell death in the hTERT and SV40 transition but not in H-Ras transition is not astonishing. Moreover, our results reflect the finding that human cells are immortalized by hTERT in combination with SV40 LT. Furthermore, such data-depiction and interpretation can give a first hint at ongoing molecular changes in an unknown dataset and enables fast data-access.

Similarly, Table 3 depicts the direct link between potentially regulated proteins and the respective transition features. Interestingly, the proteins PPME1 and SLC25A24 were identified both in the up-regulated spot 24 and within the same transition these proteins were also identified in two different down-regulated spots (spot 25 and 26). Thus, they are potentially modified due to SV40 ER expression and therefore moving in the 2D-gel spot pattern.

Focusing on the seven proteins listed in Table 4, PCNA is a commonly used proliferation marker. Within the cell culture model for malignant transformation higher proliferation is observed due to SV40 ER integration and consequently an up-regulation of PCNA after this transition was detectable with 2D-PAGE and reinforced by Western Blot. So the up-regulation of PCNA confirms the observations on cell culture growth.

PRDX6 has not been described previously to be regulated due to hTERT and SV40 ER integration. Here, we show that the integration of hTERT results in a down-regulation of the PRDX6 spot and the subsequent introduction of SV40 ER results in the complete absence of this spot. In contrast, Western Blot analysis of PRDX6 shows no difference in abundance between BJ and BJ-T, but indeed PRDX6 is clearly down-regulated due to SV40 ER and nearly absent in BJ-TER. However, another study showed the up-regulation of PRDX6 in metastatic cells [39] and, moreover, PRDX6 was found to be up-regulated in sera of many patients with esophageal squamous cell carcinoma [40]. Again, we can only speculate that the direction of regulation is influenced by the genetic background and tissue origin of the cells. Nevertheless, PRDX6 is involved in the redox regulation of the cell and may play a role in the regulation of phospholipid turnover as well as in protection against oxidative injury [41]. Changes in redox regulation and pathways appear to be important for tumorigenesis [42] so that the role of PRDX6 during tumorigenesis should to be further investigated.

PAI-2 is well documented as an inhibitor of the urokinase-type plasminogen activator [43]. Furthermore, PAI-2 has been shown to carry out a number of intracellular functions: it can alter gene expression, influence the rate of cell proliferation and differentiation, and inhibit apoptosis independent of urokinase inhibition [44]. The crucial change caused by the hTERT transition is yet unclear, but recent findings suggest that the urokinase plasminogen activator system is causally involved at multiple steps in cancer progression [45]. Interestingly, the nuclear-located PAI-2 was shown to bind to RB via its CD-loop [46].

**Figure 7:**
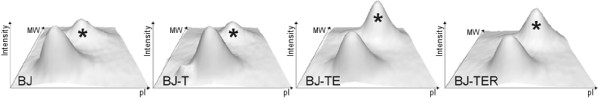
**3D view of a gel section from all cell lines: TRAP-1 (spot 32) is identified in the spot marked with an asterisk**. Representative single gel sections are selected, that is, those sections closest to the mean of all five gels from one cell line.

Also, TRAP-1 was reported to interact with RB [47]. TRAP-1 is a chaperone that expresses an ATPase activity and protects cells from apoptosis by an unknown mechanism. Possibly, in tumor cells, the up-regulation of both TRAP-1 in conjunction with HSP90, which both interact with Cyclophilin-40 (CypD) maintains mitochondrial homeostasis and survival of cells. Blocking the interaction of TRAP-1 and HSP90 with CypD leads to CypD mediated mitochondrial induced cell death [48]. In addition, Kang et al. found no increase in TRAP-1 as a result of Ras-transformation. We could support this finding, because TRAP-1 is already up-regulated by means of SV40 ER expression (Figure 7). However, Kang et al. showed HSP90 up-regulation due to Ras-transformation; this could also be reinforced by our 2D-PAGE findings: We identified the homologous proteins HSP90-alpha and HSP90-beta, sharing 87% identity to each other in spot 35. Western Blot analysis indicates up-regulation of both HSP90-alpha and HSP90-beta by SV40 ER. Taken together these results suggest that not only Ras-transformation but also SV40 ER affects HSP90 levels.

HSP90 is a molecular chaperon that has ATPase activity. Its client proteins and interaction partners are several kinases and signaling molecules e.g. Akt, p53 and Hypoxia-inducible factor 1 alpha. HSP90 occupies a unique nodal role in cellular homeostasis, overseeing cell proliferation and cell-survival mechanisms [48,49].

Surprisingly, HSP90-alpha and HSP90-beta as well as PCNA were recently identified as binding partners of p16INK4a [50], suggesting that p16INK4a possibly plays a central role in several steps of tumorigenesis distinct from kinase inhibition. Moreover, the same study also reveals several cytoskeletal proteins as interacting partners of p16INK4a. Some cytoskeletal proteins were identified within our investigation, especially six in the transition from BJ-T to BJ-TE. Considering the profound changes in cell morphology in this step, reorganization of the cytoskeleton appears to be of high importance. Furthermore, among the seven unequivocally identified proteins the cytoskeletal protein CK-7 and the possibly cytoskeleton-associated protein p36 are listed. p36 may cross-link plasma membrane phospholipids with actin and the cytoskeleton and might be involved in exocytosis [51]. Both, p36 and CK-7, protein spots are regulated in the same manner and are absent after expression of SV40 ER. In contrast, CK-7 is described to be up-regulated in several cancers and tumors e.g. breast cancer [52], transitional cell papillomas and carcinomas [53].

Besides these seven unambiguously identified proteins, mass spectrometric analysis identified further potentially regulated proteins. Although these proteins need to be validated in future experiments, our findings point to intriguing interactions between the p16-RB pathway, the mitochondrial chaperone network and the cytoskeleton. In detail, SV40 LT is known to bind RB and inactivate the cell cycle inhibitor p16-RB pathway resulting in increased cell proliferation. In addition, SV40 LT also caused marked changes in cell morphology and rendered the cells more stress-resistant (data not shown). Interestingly, we observed an up-regulation of HSP90 and the RB-interacting protein TRAP-1 (HSP75), which are part of the same mitochondrial chaperone network. Specific expression of these chaperones in mitochondria of tumor cells but not normal cells has been linked to tumor cell survival by their inhibitory effect on the mitochondrial permeability transition [48]. While expression of TRAP-1 was stimulated in response to SV40 early region, mitochondrial HSP90 was induced by oncogenic Ras [48]. Up-regulation of TRAP-1 and HSP90 is therefore directly linked to defined genetic alterations and supports a model of oncogene cooperation in the establishment of this tumor-survival promoting chaperone network. Furthermore, HSP90 has also been identified together with cytoskeletal proteins in immunoprecipitates of the cell cycle inhibitor p16INK4a suggesting a possible link between HSP90 up-regulation and the changes in cytoskeleton dynamics of Ras-transformed cells [50]. The proteomic changes detected in this study therefore provide further insight into the molecular basis underlying malignant transformation. The presented list of proteins enables a more detailed analysis of specific proteins and pathways involved in cancer development. In addition, the list allows the investigation of other cell lines and even different types of cancer concerning the abundance of these proteins. We will therefore further investigate this model system.

## Abbreviations

Akt: Protein kinase B; CK-7: cytokeratin-7; CypD: Cyclophilin-40; DNA: Deoxyribonucleic acid; DTT: Dithiothreitol; ESI: electrospray ionization; GO: Gene Ontology; HSP: heat shock protein; hTERT: catalytic subunit of the telomerase holoenzyme (Telomerase reverse transcriptase); IEF: isoelectric focusing; IPG: immobilized pH gradient; IPI: international protein index; iTRAQ: isobaric tag for relative and absolute quantitation; LC: liquid chromatography; LT: large tumor antigen; MALDI: matrix-assisted laser desorption/ionization; MEFs: mouse embryonic fibroblasts; MS: mass spectrometry; MS/MS: tandem mass spectrometry; MW: molecular weight; p16INK4a: Cyclin-dependent kinase inhibitor 2A; p36: Annexin A2; p53: Tumor suppressor p53; PAGE: polyacrylamide gelelectrophorese; 2D-PAGE: two-dimensional PAGE; PAI-2: Plasminogen activator inhibitor 2; PBS: phosphate buffered saline; PCNA: proliferating cell nuclear antigen; pI: isoelectric point; PP2A: Serin/threonine-protein phosphatase; PRDX6: Peroxiredoxin-6; RB: Retinoblastoma protein; RuBPS: Ruthenium(II)tris-bathophenanthroline disulfonate; ST: small tumor antigen; SV40: simian virus 40; SV40 ER: simian virus 40 early region; TRAP-1: mitochondrial heat shock protein 75 kDa.

## Competing interests

The authors declare that they have no competing interests.

## Authors' contributions

SP made conception of the study, carried out acquisition, analysis and interpretation of data and wrote the manuscript. FV made the Western Blot experiments. TS provided the cell lines. TS and AS participated in the design of the study and contributed to the manuscript. All authors read and approved the final manuscript.

## Supplementary Material

Additional file 1 **Detailed information on the analysed 2D-gels and identified proteins**. The first page of the Additional_file_1.pdf lists identified proteins within the protein spots showing at least two-fold up- or down-regulation between the cell lines of the malignant transformation model. Shown are the Isoelectric point (pI), molecular weight (MW), maximal sequence coverage (seq.cov), maximal identified peptides (ident. peptides), and the maximal score (score) of the proteins. The regulation of the protein spot relating to BJ and their normalized standard deviation (±) is specified. Moreover, on the following pages all 2D-Gels of the analysis are shown. Click here for file
