# Supplementary material for: Malignant transformation in a defined genetic background: proteome changes displayed by 2D-PAGE
Source: Mol Cancer. 2010 Sep 22;9:254. doi: 10.1186/1476-4598-9-254 (PMC2955615; doi:10.1186/1476-4598-9-254)
Supplement: Additional file 1 — Detailed information on the analysed 2D-gels and identified proteins. The first page of the Additional_file_1.pdf lists identified proteins within the protein spots showing at least two-fold up- or down-regulation between the cell lines of the malignant transformation model. Shown are the Isoelectric point (pI), molecular weight (MW), maximal sequence coverage (seq.cov), maximal identified peptides (ident. peptides), and the maximal score (score) of the proteins. The regulation of the protein spot relating to BJ and their normalized standard deviation (±) is specified. Moreover, on the following pages all 2D-Gels of the analysis are shown. [file 1476-4598-9-254-S1.PDF]

spot no	protein ID	protein	gene	pI	MW	seq. cov	ident. peptides	score	BJ	±	T	±	TE	±	TER	±
**1**	P04792	Heat-shock protein beta-1	HSPB1	5,98	22768	57	13	791	1.00	0.15	0.69	0.30	0.17	0.03	0.13	0.05
	Q13242	Splicing factor, arginine/serine-rich 9	SFRS9	8.74	25526	14	3	164								
**2**	P30041	Peroxiredoxin-6	PRDX6	6,02	24888	72	19	1261	1.00	0.26	0.25	0.05	0.00	0.00	0.00	0.00
**3**	Q06323	Proteasome activator complex subunit 1	PSME1	5.78	28705	45	15	767	1.00	0.19	0.98	0.36	1.03	0.09	2.05	0.59
	P35232	Prohibitin	PHB	5,57	29786	21	6	290								
**4**	Q9UNF0	Protein kinase C and casein kinase substrate in neurons protein 2	PACSIN2	5.08	55704	15	8	457	1.00	0.21	1.10	0.35	0.40	0.09	0.34	0.02
**5**	P43487	Ran-specific GTPase-activating protein	RANBP1	5.19	23296	34	6	349	1.00	0.43	0.79	0.26	2.18	0.47	2.61	0.84
	P25788	Proteasome subunit alpha type 3	PSMA3	5.19	28284	45	3	664								
	P07686	Beta-hexosaminidase beta chain precursor	HEXB	6.29	63071	14	3	461								
**6**	Q15691	APC-binding protein EB1	MAPRE1	5,02	29849	46	11	529	1.00	0.29	1.03	0.31	2.46	0.76	1.69	0.46
	Q99426	Tubulin-specific chaperone B	TBCB	5,06	27308	7	2	88								
	P61247	40S ribosomal protein S3a	RPS3A	9,75	29795	11	3	125								
**7**	N.D.								1.00	0.73	1.99	1.40	5.53	1.57	5.98	0.90
**8**	P12004	Proliferating cell nuclear antigen	PCNA	4,57	28750	62	13	936	1.00	0.29	1.10	0.36	2.59	0.45	2.97	0.79
	P01584	Interleukin-1 beta precursor	IL1B	4,70	30728	9	2	126								
**9**	N.D.								1.00	0.27	0.88	0.13	0.27	0.08	0.20	0.10
**10**	N.D.								1.00	0.12	0.76	0.12	0.21	0.08	0.16	0.09
**11**	O00463	TNF receptor-associated factor 5	TRAF5	7,26	64364	1	2	91	1.00	0.25	0.71	0.07	0.30	0.09	0.16	0.05
**12**	P13716	Delta-aminolevulinic acid dehydratase	ALAD	6.32	36271	22	6	394	1.00	0.18	0.72	0.18	0.48	0.05	0.44	0.04
	O14656	Torsin A precursor	TOR1A	6.51	37784	16	5	371								
**13**	P07355	Annexin A2	ANXA2	7.56	38449	57	20	1210	1.00	0.30	2.58	1.04	0.00	0.00	0.00	0.00
**14**	P31942	Heterogeneous nuclear ribonucleoprotein H3	HNRPH3	6.37	36903	17	5	379	1.00	0.16	0.99	0.11	0.29	0.07	0.18	0.04
	P02545	Lamin-A/C	LMNA	6.57	74095	21	14	859								
**15**	N.D.								1.00	0.18	0.62	0.04	0.39	0.09	0.40	0.10
**16**	N.D.								1.00	0.12	0.95	0.12	0.41	0.09	0.34	0.05
**17**	O60664	Mannose-6-phosphate receptor-binding protein 1	M6PRBP1	5.30	47018	17	6	419	1.00	0.43	2.30	0.15	3.26	0.63	2.40	0.88
	P06748	Nucleophosmin	NPM1	4.64	32555	17	4	199								
	P07195	L-lactate dehydrogenase B chain	LDHB	5.72	36484	20	7	397								
**18**	P08670	Vimentin	VIM	5,06	53488	23	11	712	1.00	0.32	1.03	0.16	0.33	0.20	0.23	0.13
**19**	Q9UJZ1	Stomatin-like protein 2	STOML2	6.88	38510	56	19	1372	1.00	0.42	1.24	0.26	2.46	0.38	2.81	0.57
	Q9NWT6	Hypoxia-inducible factor 1 alpha inhibitor	HIF1AN	5.39	40260	18	7	471								
	O94905	SPFH domain-containing protein 2 precursor	ERLIN2	5.47	37815	17	5	397								
**20**	P05120	Plasminogen activator inhibitor 2 precursor	SERPINB2	5.46	46566	50	21	2390	1.00	0.47	2.18	0.56	1.84	0.29	1.50	0.21
	P60709	Beta-actin	ACTB	5.29	41710	33	11	646								
**21**	P60709	Beta-actin	ACTB	5.29	41710	19	8	426	1.00	0.16	1.09	0.09	0.53	0.05	0.38	0.06
**22**	P60709	Beta-actin	ACTB	5.29	41710	26	10	602	1.00	0.08	1.80	0.42	0.00	0.00	0.39	0.02
	Q12905	Interleukin enhancer-binding factor 2	ILF2	5.19	43035	23	7	490								
	P60842	Eukaryotic initiation factor 4A-I	EIF4A1	5.32	46125	22	8	485								
	O60664	Mannose-6-phosphate receptor-binding protein 1	M6PRBP1	5.30	47018	7	3	200								
**23**	N.D.								1.00	0.50	2.14	0.98	0.42	0.31	0.36	0.00
**24**	Q9Y570	Protein phosphatase methylesterase 1	PPME1	5.67	42157	12	4	179					235347	53.60%	119221	39.90%
	IPI00337494	Solute carrier family 25 member 24	SLC25A24	6	53320	35	17	850								
**25**	Q9Y570	Protein phosphatase methylesterase 1	PPME1	5,67	42157	37	9	1085	1.00	0.33	0.79	0.17	0.20	0.08	0.16	0.07
	Q13148	TAR DNA-binding protein 43	TARDBP	5.85	44711	18	3	391								
	P60709	Beta-actin	ACTB	5.29	41710	19	7	397								
**26**	P23526	Adenosylhomocysteinase	AHCY	5.92	47554	7	3	192	1.00	0.13	0.85	0.11	0.40	0.08	0.33	0.09
	P36507	Dual specificity mitogen-activated protein kinase kinase 2	MAP2K2	6.12	44396	26	2	625								
	IPI00410404	thymidylate kinase family LPS-inducible member	none	6.57	49417	24	10	681								
	IPI00337494	Solute carrier family 25 member 24	SLC25A24	6	53320	32	15	857								
**27**	P06733	Alpha-enolase	ENO1	6.99	47008	25	9	586	1.00	0.22	1.07	0.15	0.57	0.00	0.00	0.00
	P68104	Elongation factor 1-alpha 1	EEF1A1	9,10	50109	4	2	96								
**28**	P09913	Interferon-induced 54 kDa protein	IFIT2	6.32	54597	36	18	1141	1.00	0.09	0.79	0.09	0.06	0.02	0.08	0.02
	P00352	Aldehyde dehydrogenase, cytosolic	ALDH1A1	6.29	54696	46	25	1732								
	Q15813	Tubulin-specific chaperone E	TBCE	6.32	59309	23	14	884								
	P12268	Inosine-5'-monophosphate dehydrogenase 2	IMPDH2	6.44	55770	25	12	742								
	O43175	D-3-phosphoglycerate dehydrogenase	PHGDH	6.31	56483	16	8	574								
	P11413	Glucose-6-phosphate 1-dehydrogenase	G6PD	6.44	59097	16	9	452								
**29**	P00352	Aldehyde dehydrogenase, cytosolic	ALDH1A1	6,29	54696	42	21	1296	1.00	0.35	0.71	0.21	0.00	0.00	0.00	0.00
	O43175	D-3-phosphoglycerate dehydrogenase	PHGDH	6,31	56483	21	9	606								
	Q9UMS4	Pre-mRNA splicing factor 19	PRPF19	6,14	55146	9	5	290								
**30**	P11413	Glucose-6-phosphate 1-dehydrogenase	G6PD	6.44	59097	6	4	197	1.00	0.29	0.74	0.26	0.00	0.00	0.00	0.00
	P28838	Cytosol aminopeptidase	LAP3	6.29	52607	9	4	245								
**31**	N.D.								1.00	0.89	0.49	0.31	0.23	0.00	0.00	0.00
**32**	Q12931	Heat shock protein 75 kDa, mitochondrial precursor	TRAP1	8,30	80060	38	24	1468	1.00	0.32	1.36	0.24	3.16	0.82	2.15	0.52
**33**	P06396	Gelsolin precursor	GSN	5.9	85644	37	23	1548	1.00	0.18	0.88	0.15	0.36	0.12	0.48	0.11
	Q96TA1	Niban-like protein	FAM129B	5.81	82631	16	14	848								
**34**	P20591	Interferon-induced GTP-binding protein Mx1	MX1	5.6	75356	71	41	2081	1.00	0.29	1.78	0.27	1.59	0.59	2.10	0.84
	P13667	Protein disulfide-isomerase A4 precursor	PDIA4	4.96	72887	43	31	1789								
	P11021	78 kDa glucose-regulated protein precursor	HSPA5	5.07	72288	14	8	492								
	P17812	CTP synthase	CTPS	6.02	66648	15	11	599								
**35**	P08238	Heat shock protein HSP 90-beta	HSP90AB1	4,97	83081	51	45	2582	1.00	0.93	0.99	0.64	0.97	0.67	2.07	1.12
	P07900	Heat shock protein HSP 90-alpha	HSP90AA1	4,94	84490	38	36	2042								
**36**	P30048	Thioredoxin-dependent peroxide reductase, mitochondrial precursor	PRDX3	7.67	27675	33	7	687	1.00	0.44	0.94	0.43	1.86	0.31	2.07	1.05
	P62491	Ras-related protein Rab-11A	RAB11A	6.14	24247	40	10	455								
	P27635	60S ribosomal protein L10	RPL10	10.11	24430	34	9	310								
**37**	P10809	60 kDa heat shock protein, mitochondrial precursor	HSPD1	5.70	61016	61	32	2106	1.00	0.48	1.10	0.28	1.79	0.66	2.09	0.59
	P68363	Tubulin alpha-ubiquitous chain	K-ALPHA-1	4.94	50120	18	8	362								
	P07237	Protein disulfide-isomerase precursor	P4HB	4.76	57081	34	17	846								
	P60709	Beta-actin	ACTB	5.29	41710	24	8	368								
	P54578	Ubiquitin carboxyl-terminal hydrolase 14	USP14	5.20	55902	15	7	357								
	P48643	T-complex protein 1 subunit epsilon	CCT5	5.45	59633	1	1	78								
**38**	P08729	Keratin, type II cytoskeletal 7	KRT7	5.23	51418	60	40	2559	1.00	0.08	0.96	0.12	0.00	0.00	0.00	0.00
**39**	Q969P6	DNA topoisomerase I, mitochondrial precursor	TOP1MT	9,46	69828	1	1	50	1.00	0.26	0.85	0.11	0.43	0.06	0.38	0.09
	P08670	Vimentin	VIM	5,06	53488	57	27	1765								
	P14625	Endoplasmin precursor	HSP90B1	4.76	92411	8	6	369								

Supplemental table 1

Identified proteins within the protein spots showing at least two-fold up- or down-regulation between the cell lines of the malignant transformation model are listed. Shown are the Isoelectric point (pI), molecular weight (MW), maximal sequence coverage (seq.cov), maximal identified peptides (ident. peptides), maximal score (score) of the proteins. The regulation of the protein spot relating to BJ which was normalized to 1 and their normalized standard deviation (±) is specified.

BJ_01

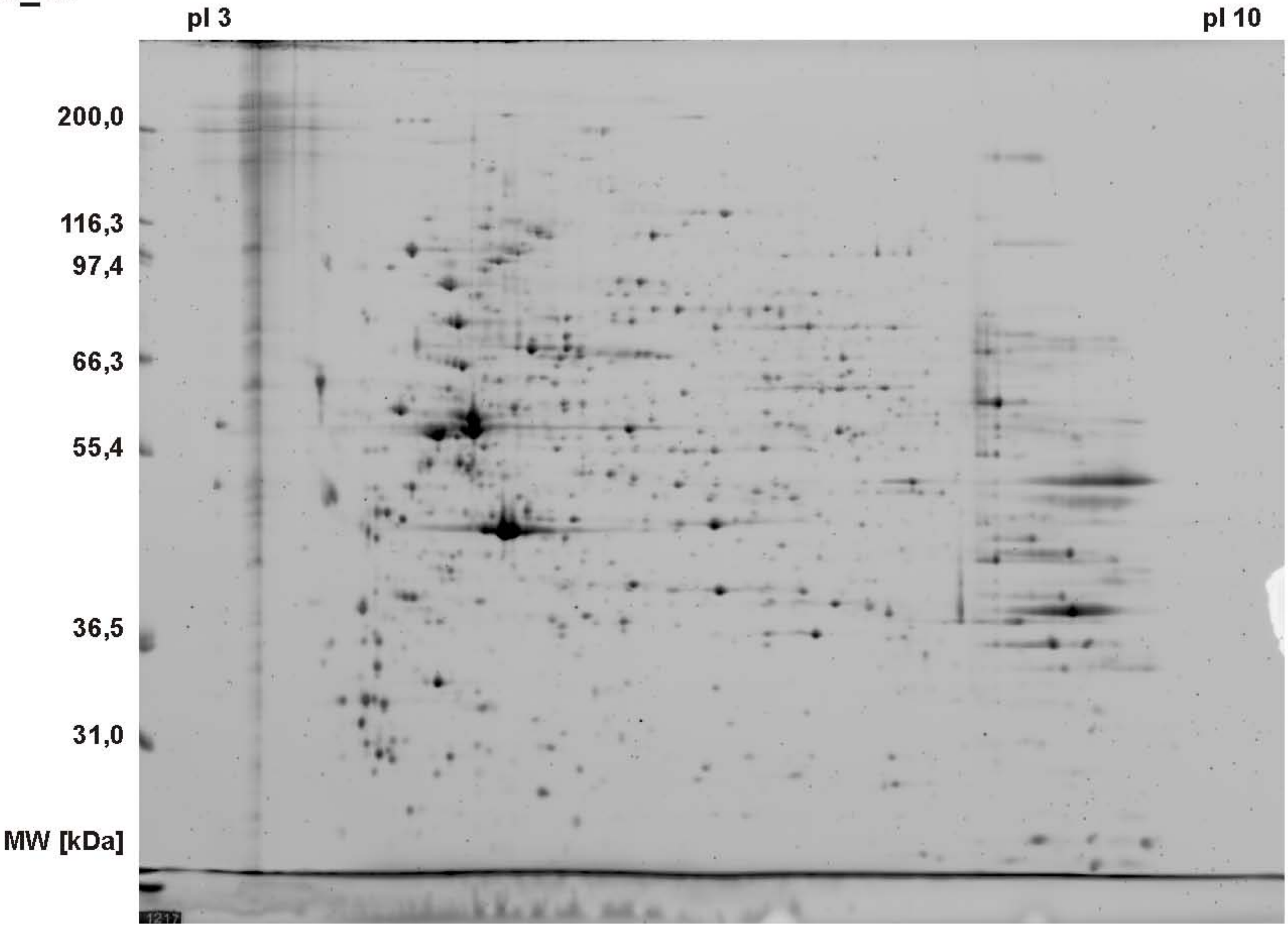

BJ_02

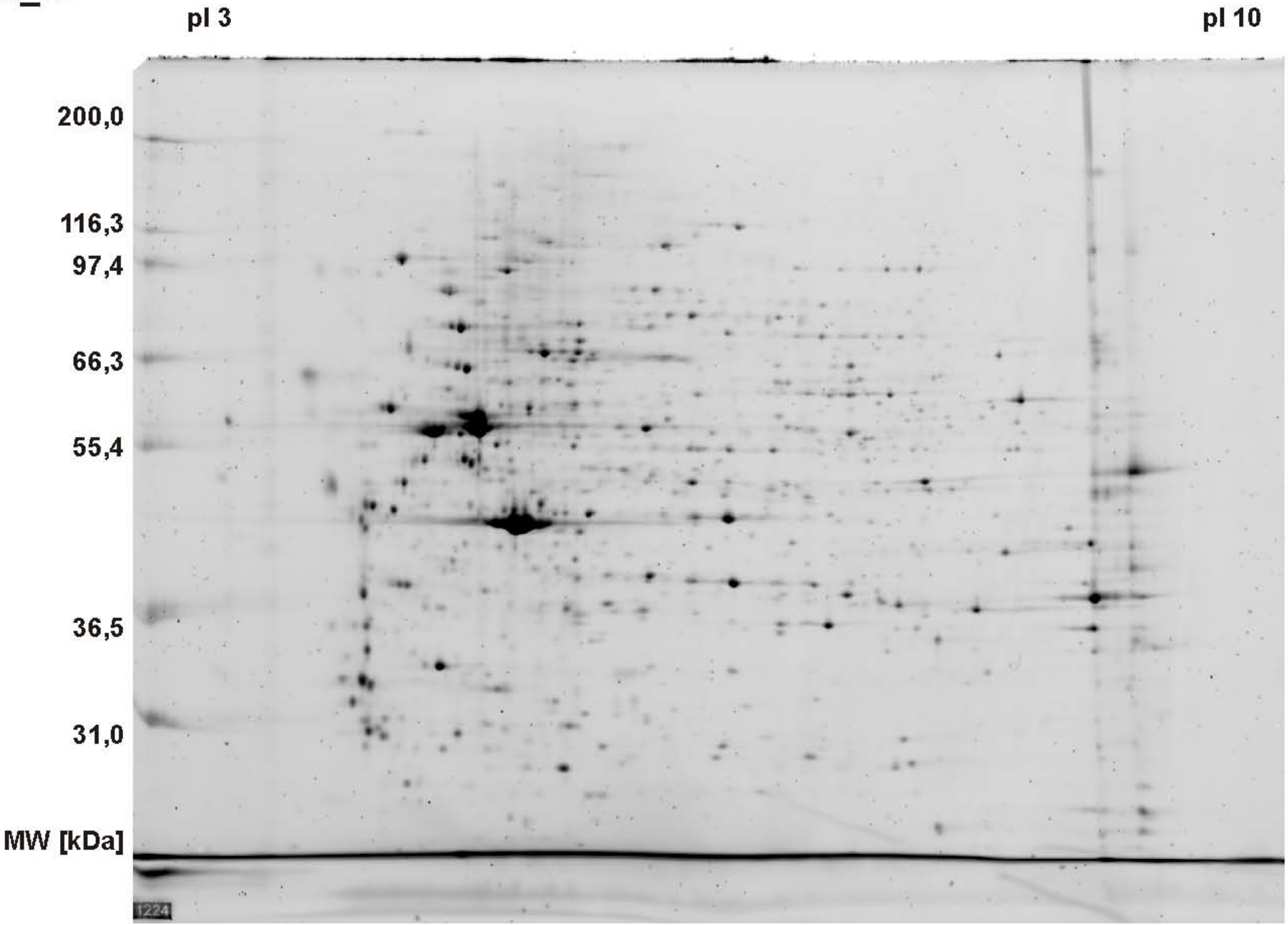

BJ_03

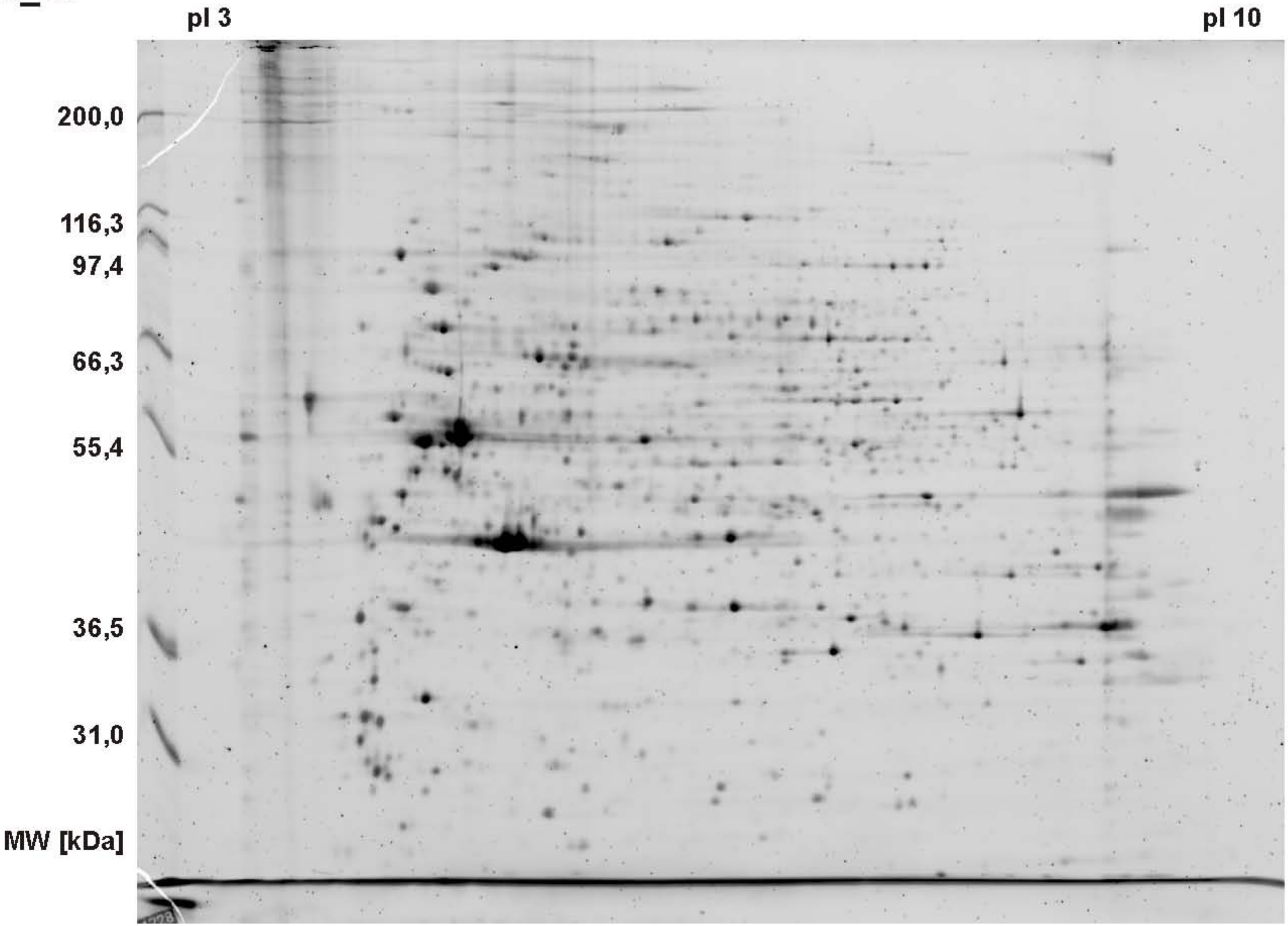

BJ_04

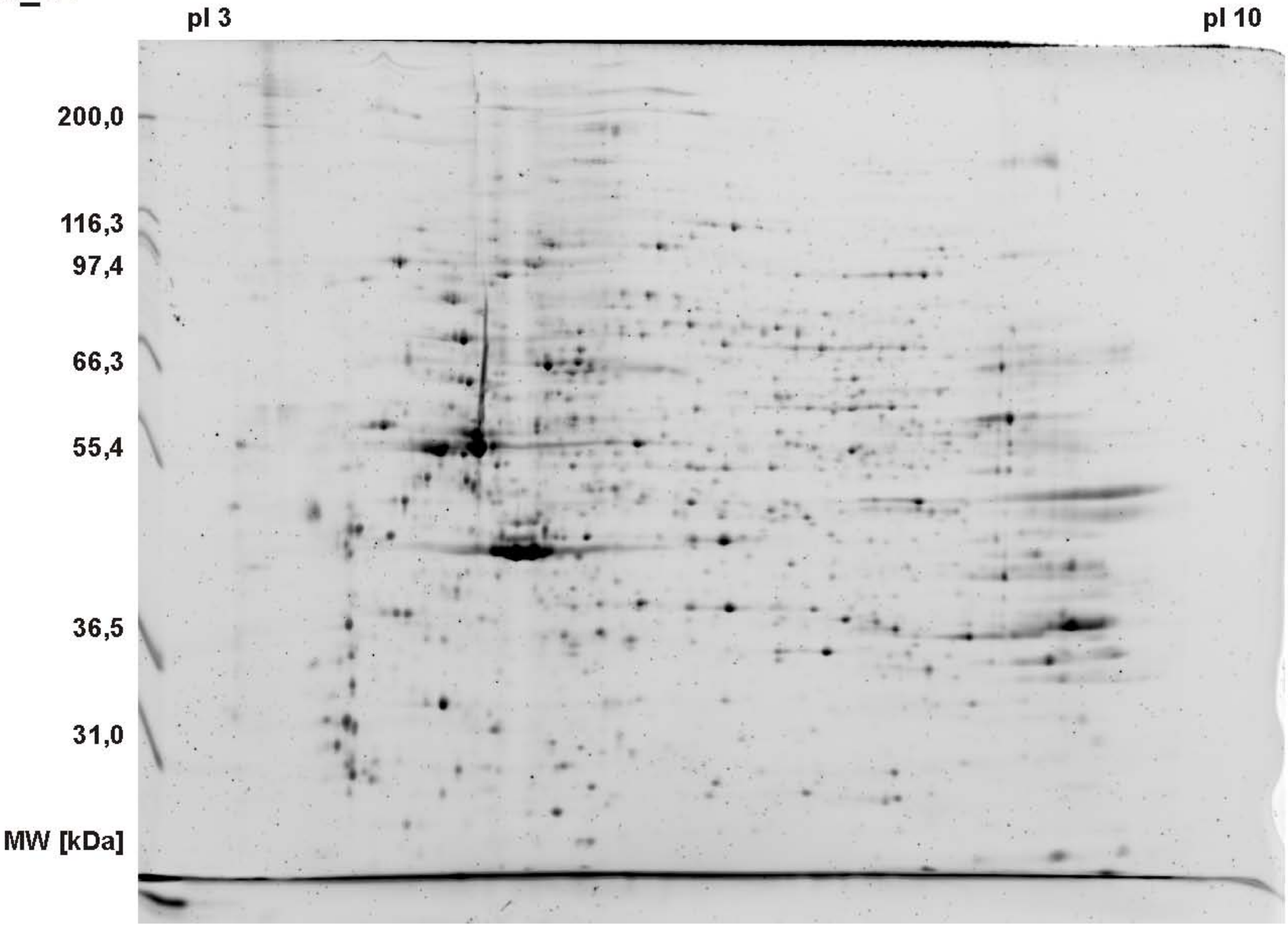

BJ_05

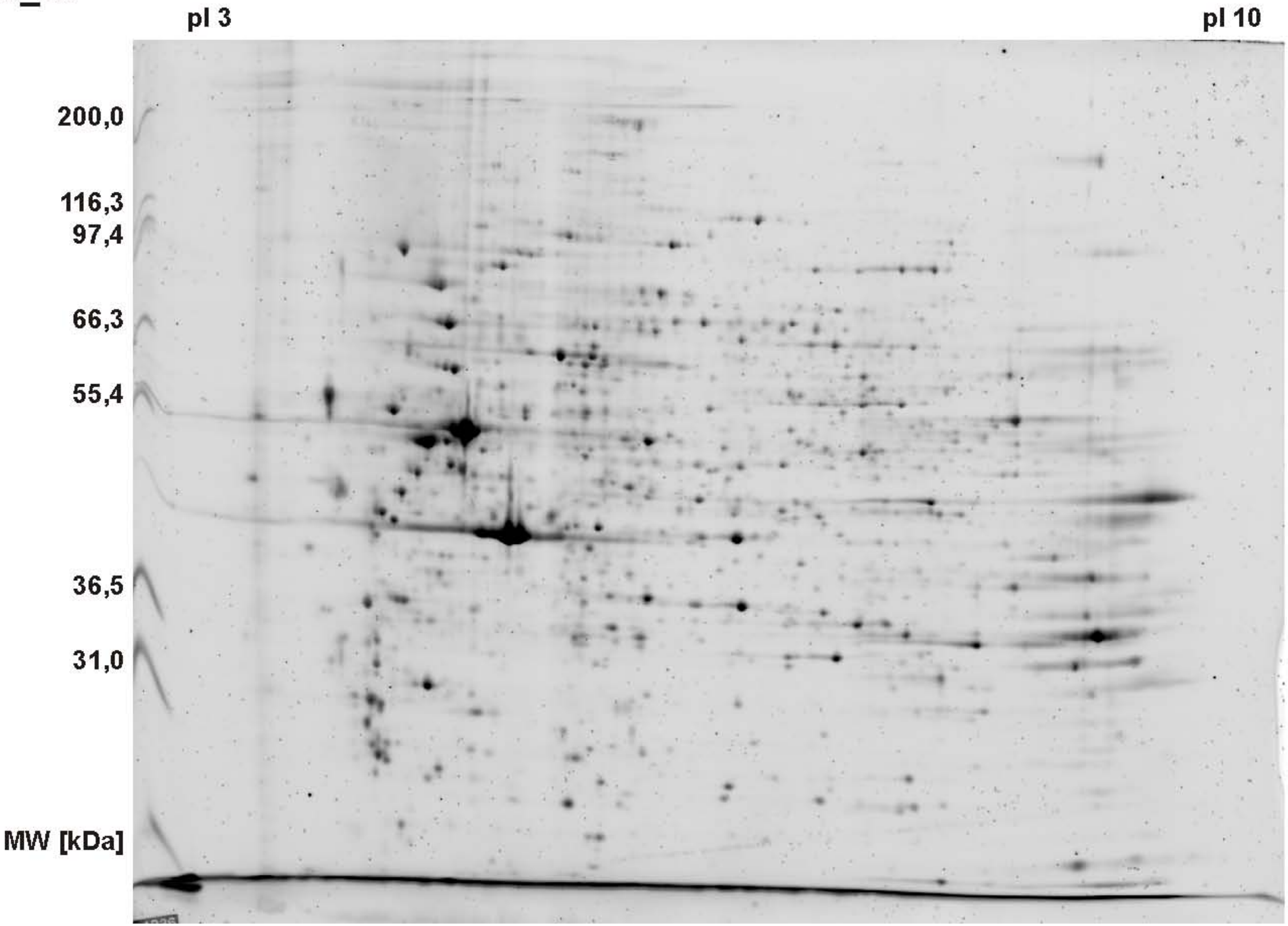

BJ-T_01

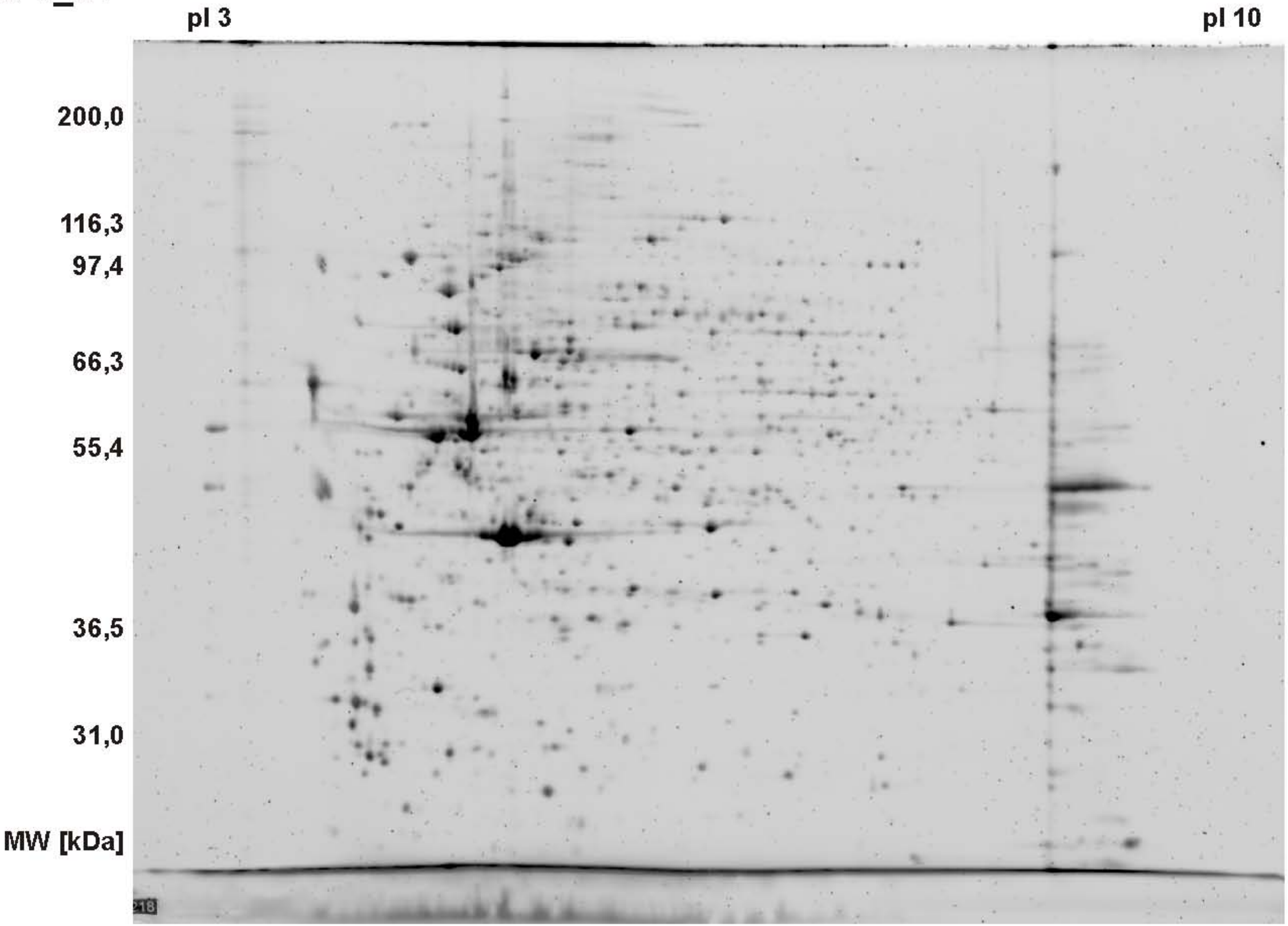

BJ-T_02

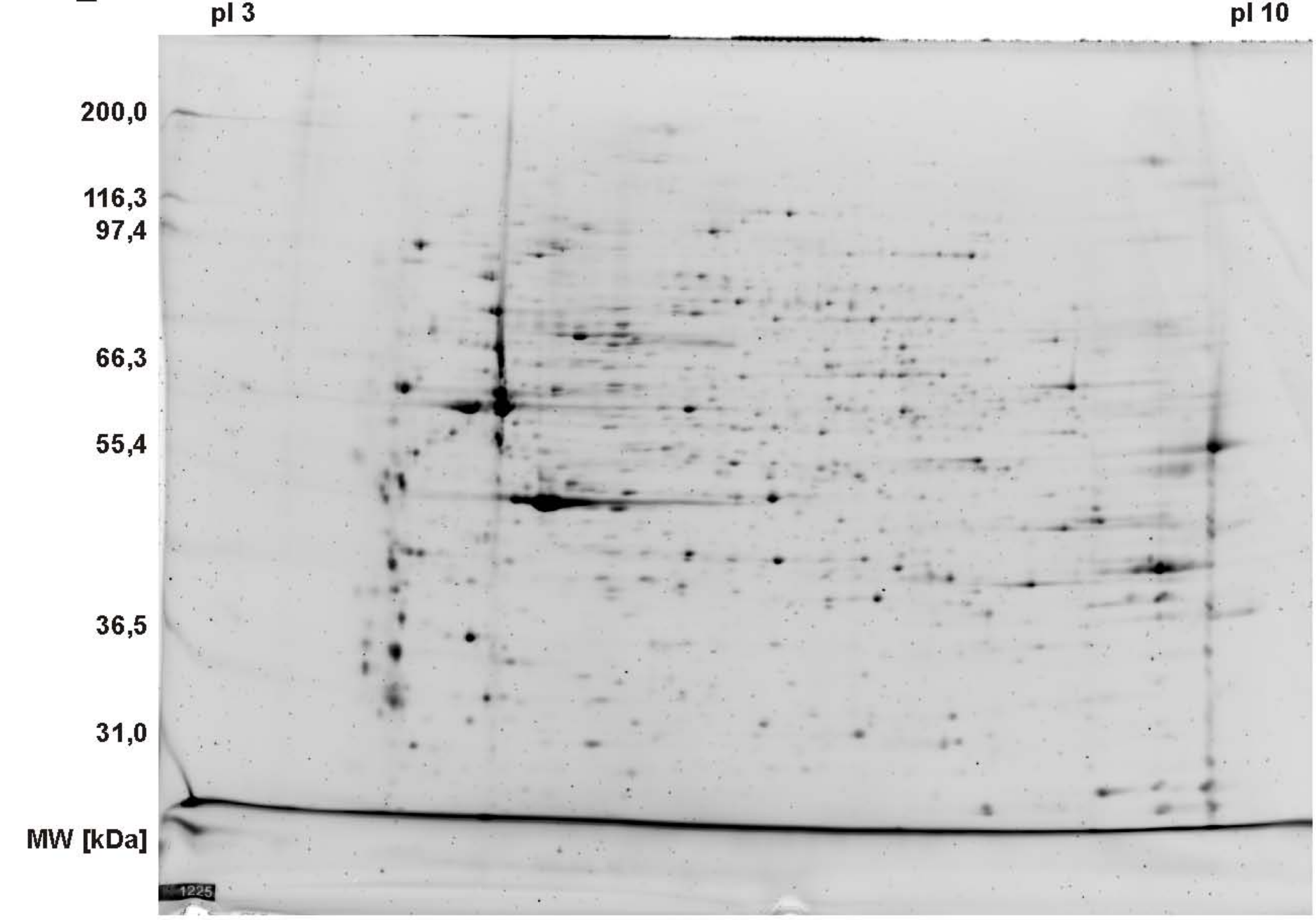

BJ-T_03

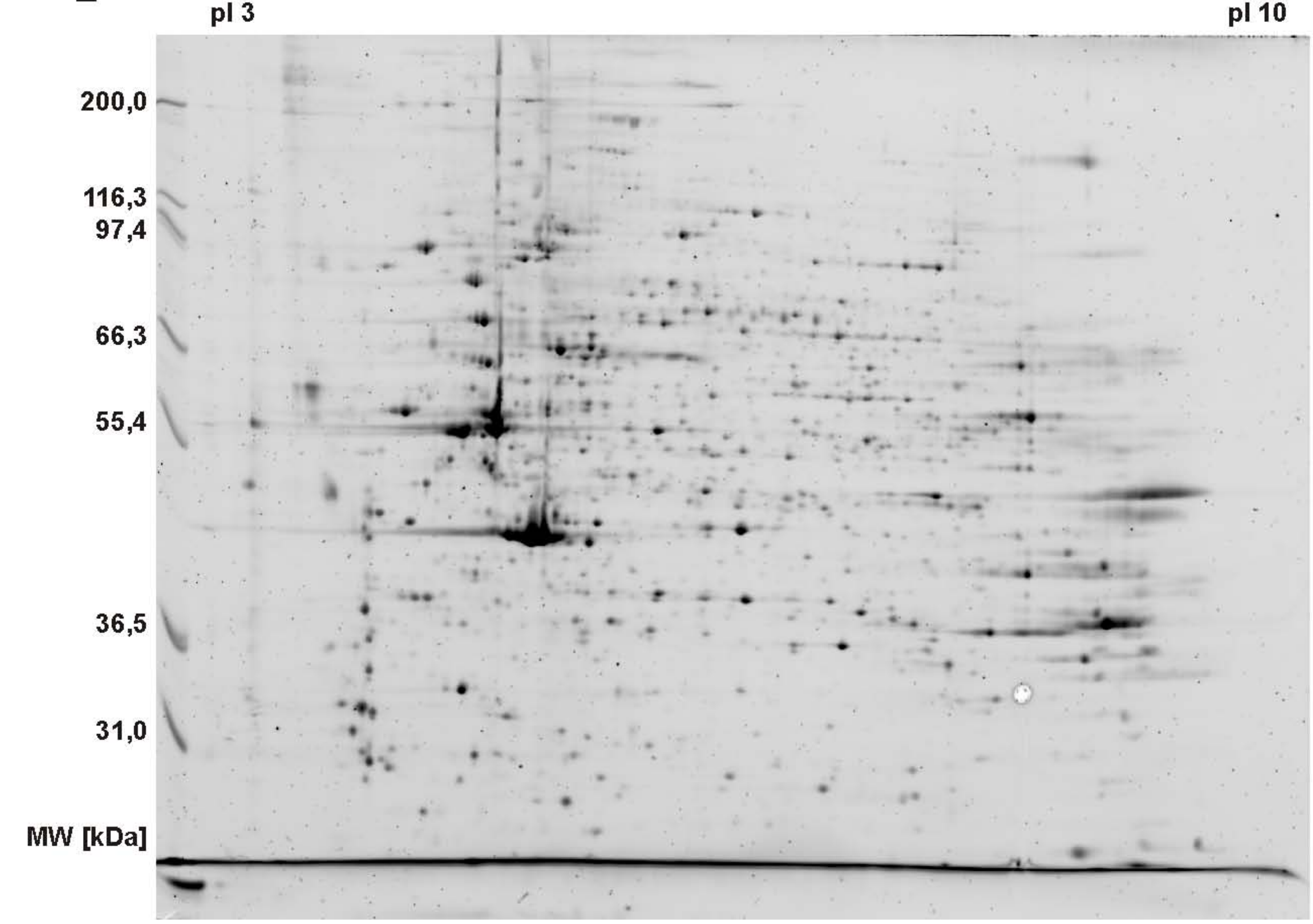

BJ-T_04

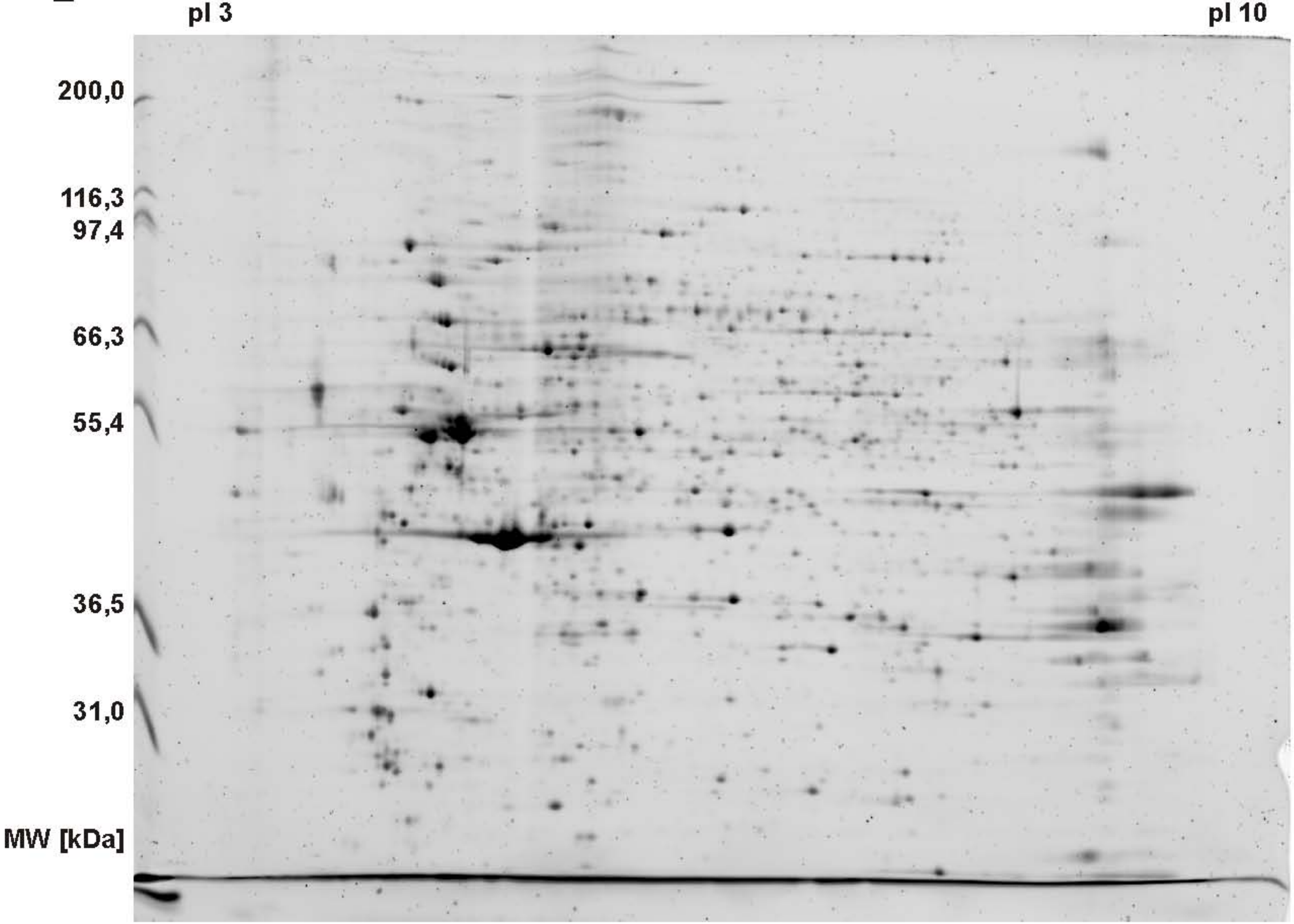

BJ-T_05

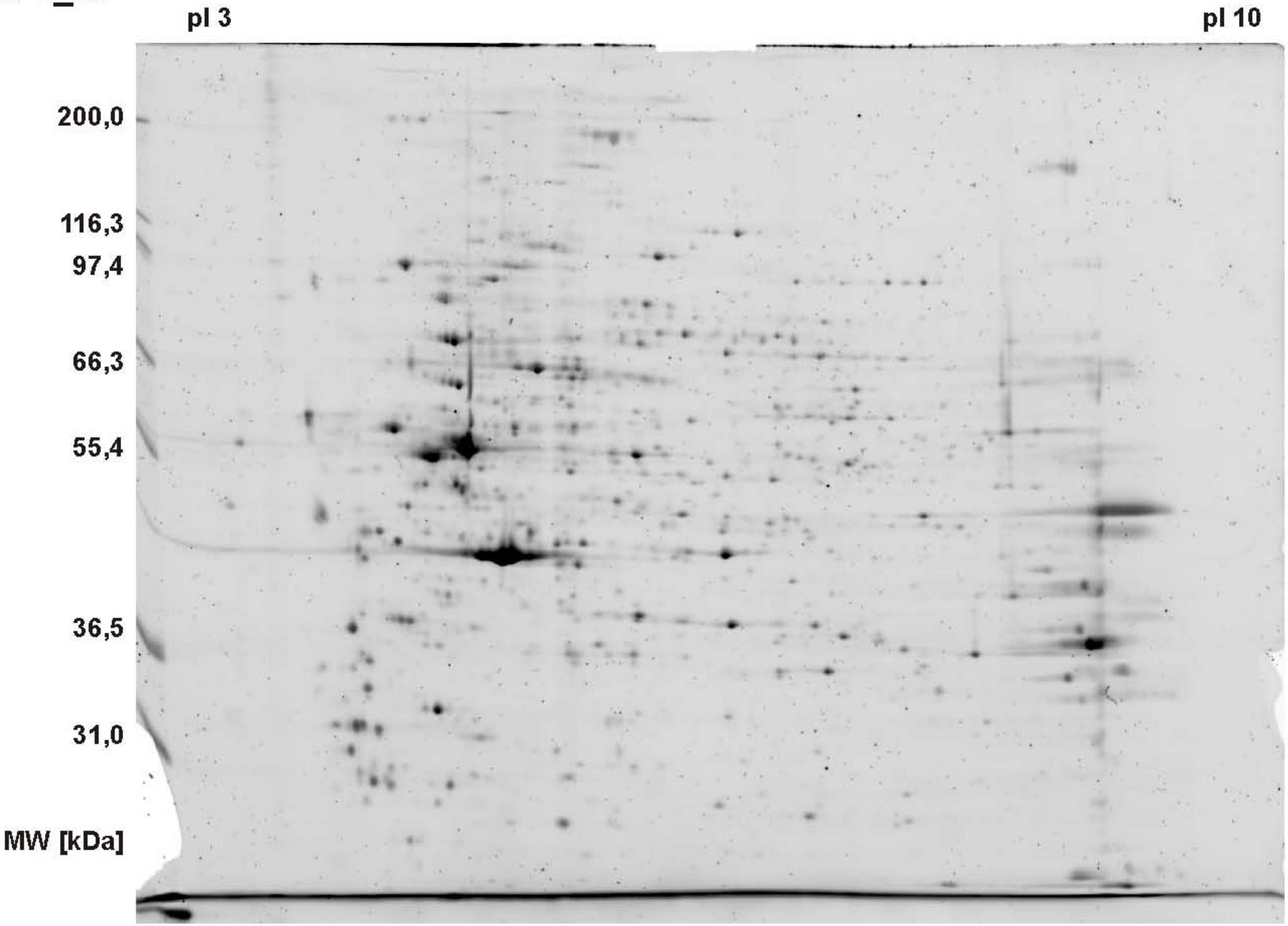

BJ-TE_01

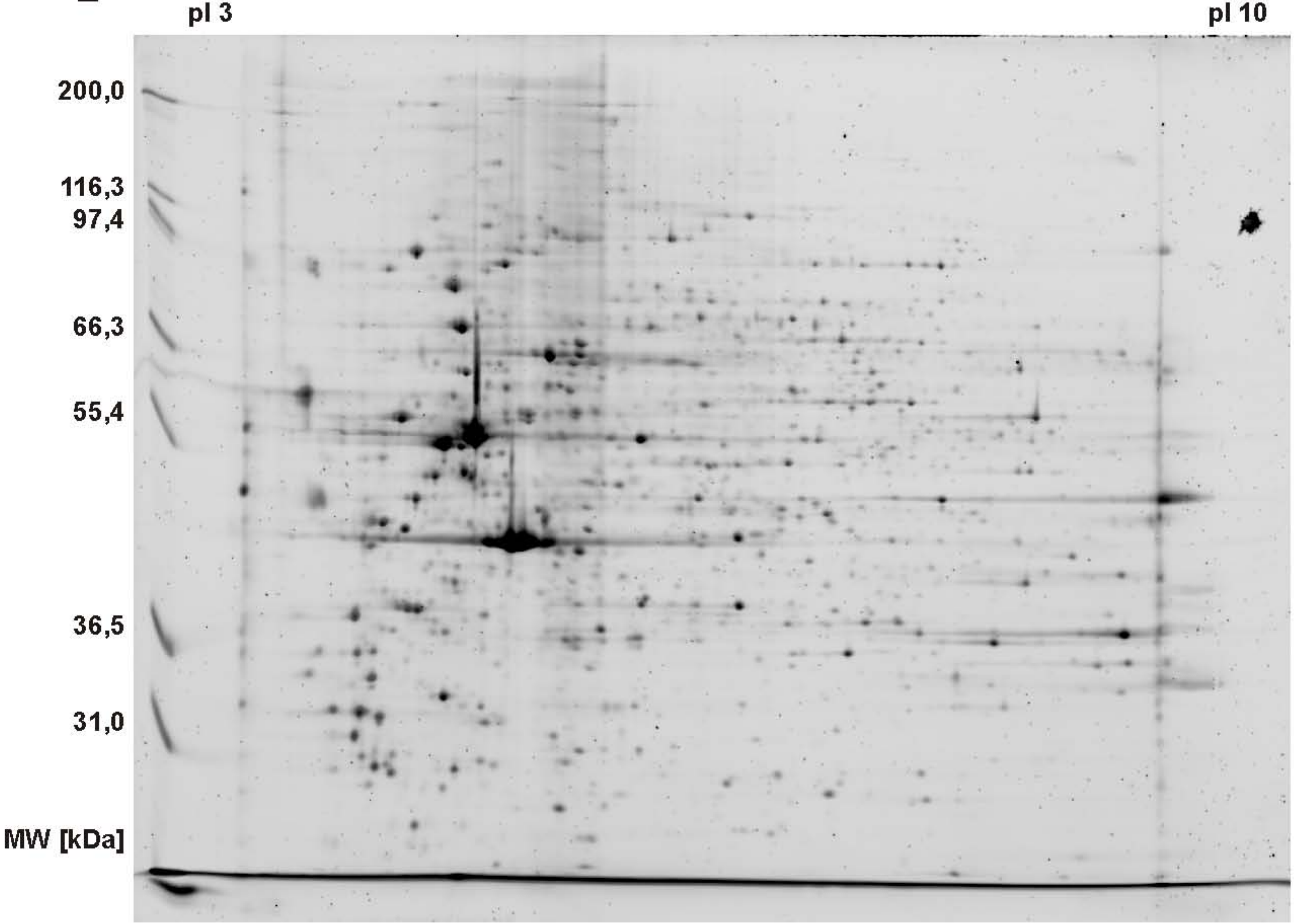

BJ-TE_02

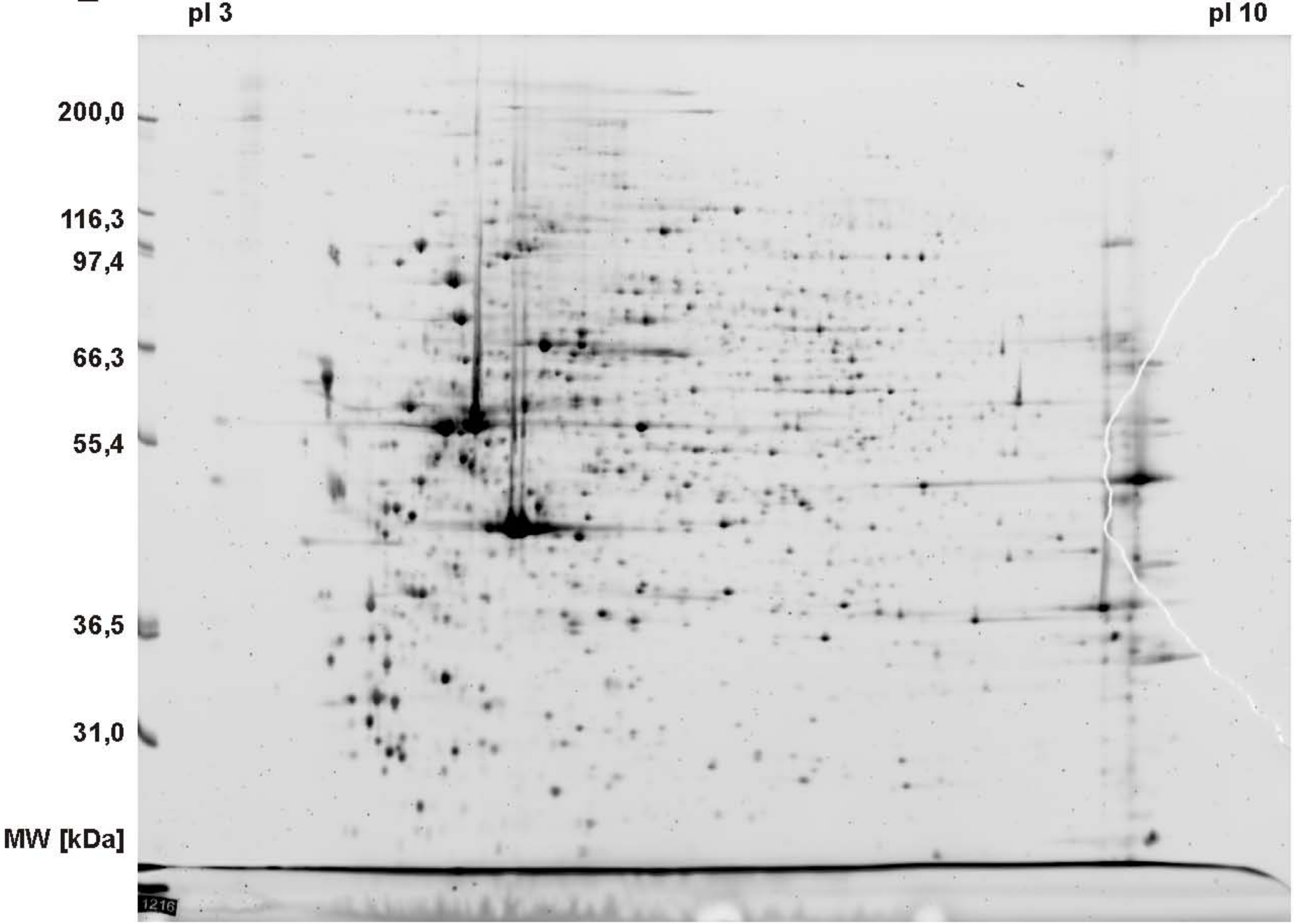

BJ-TE_03

pI 3 pI 10

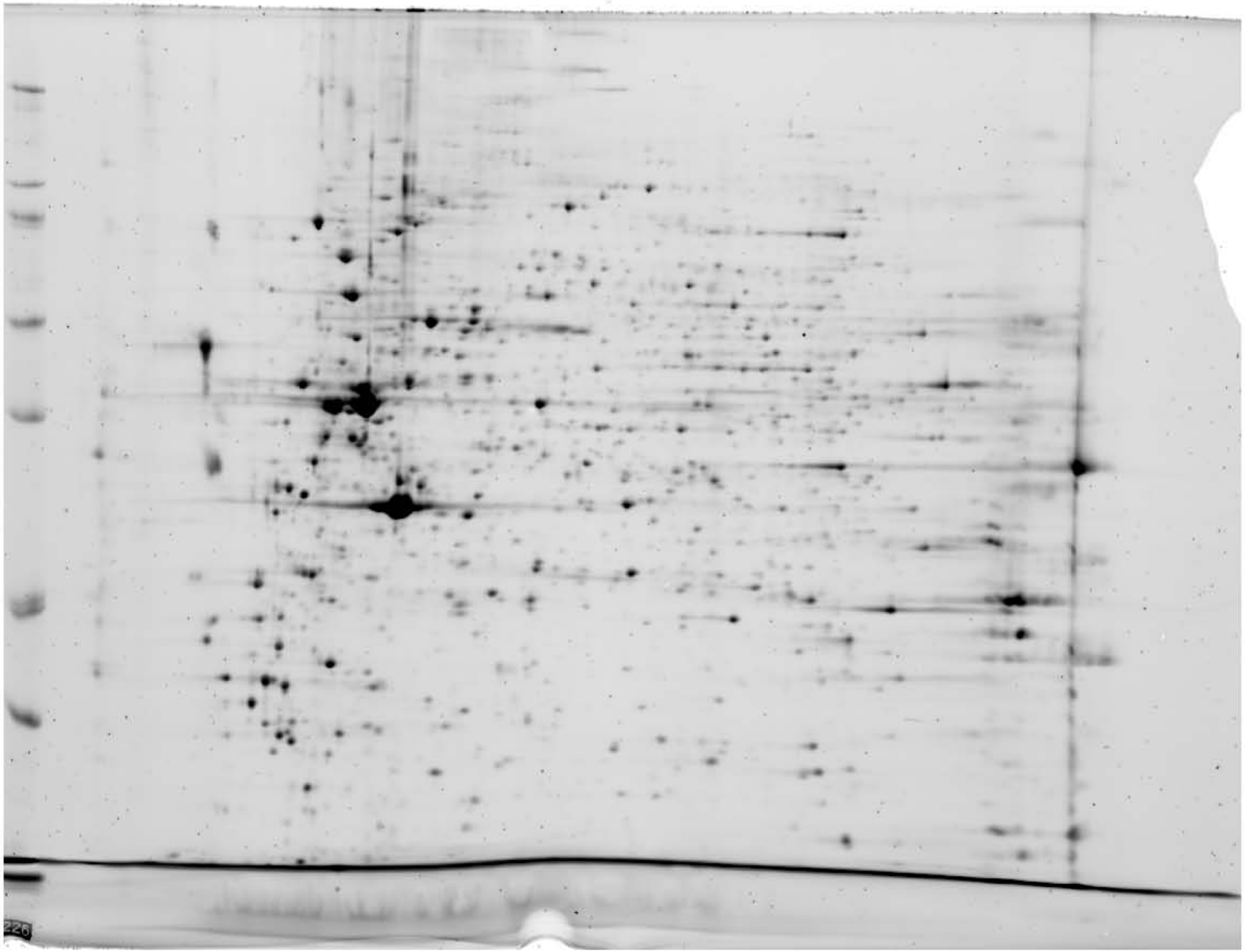

200,0
116,3
97,4
66,3
55,4
36,5
31,0
MW [kDa]

BJ-TE_04

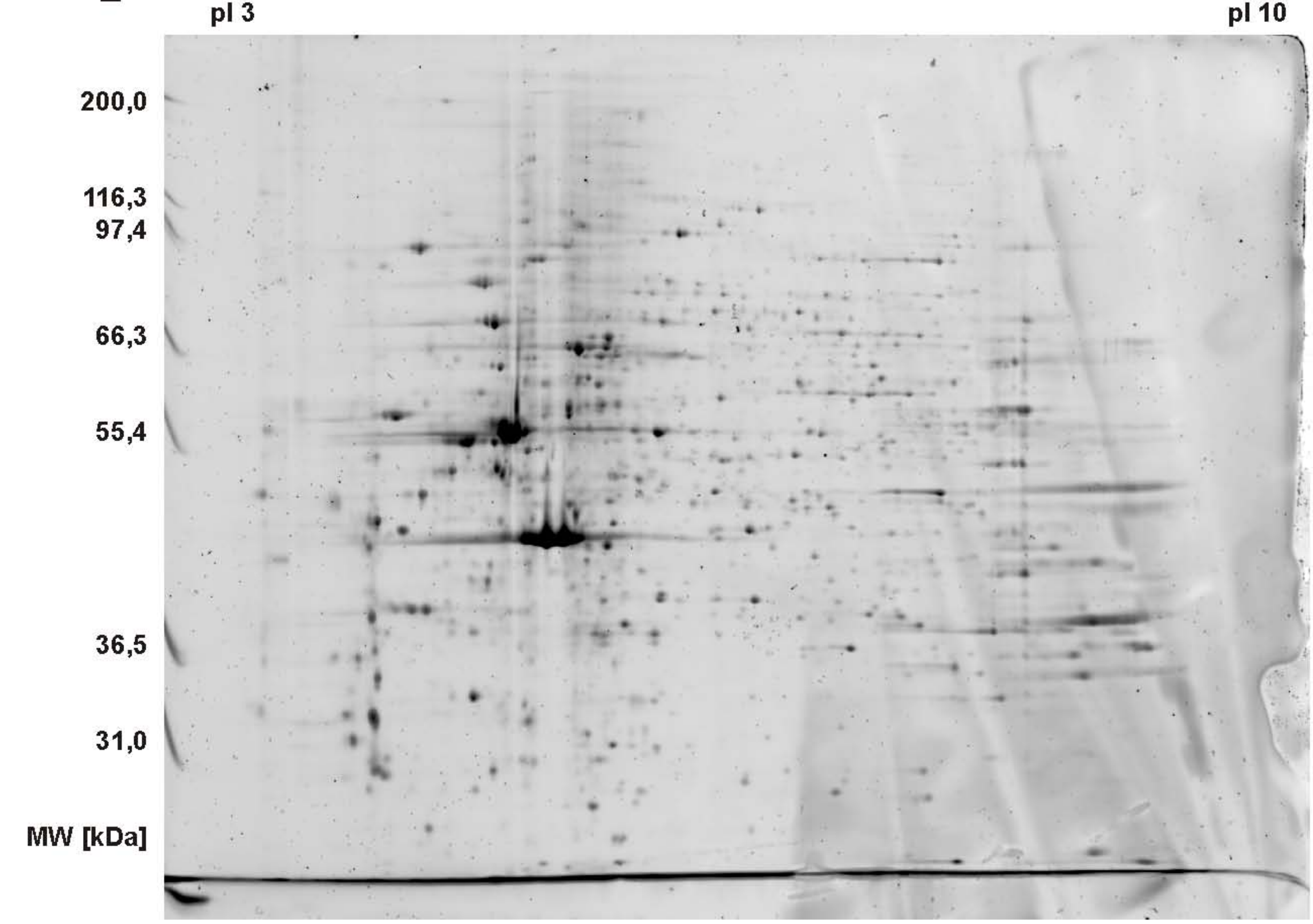

BJ-TE_05

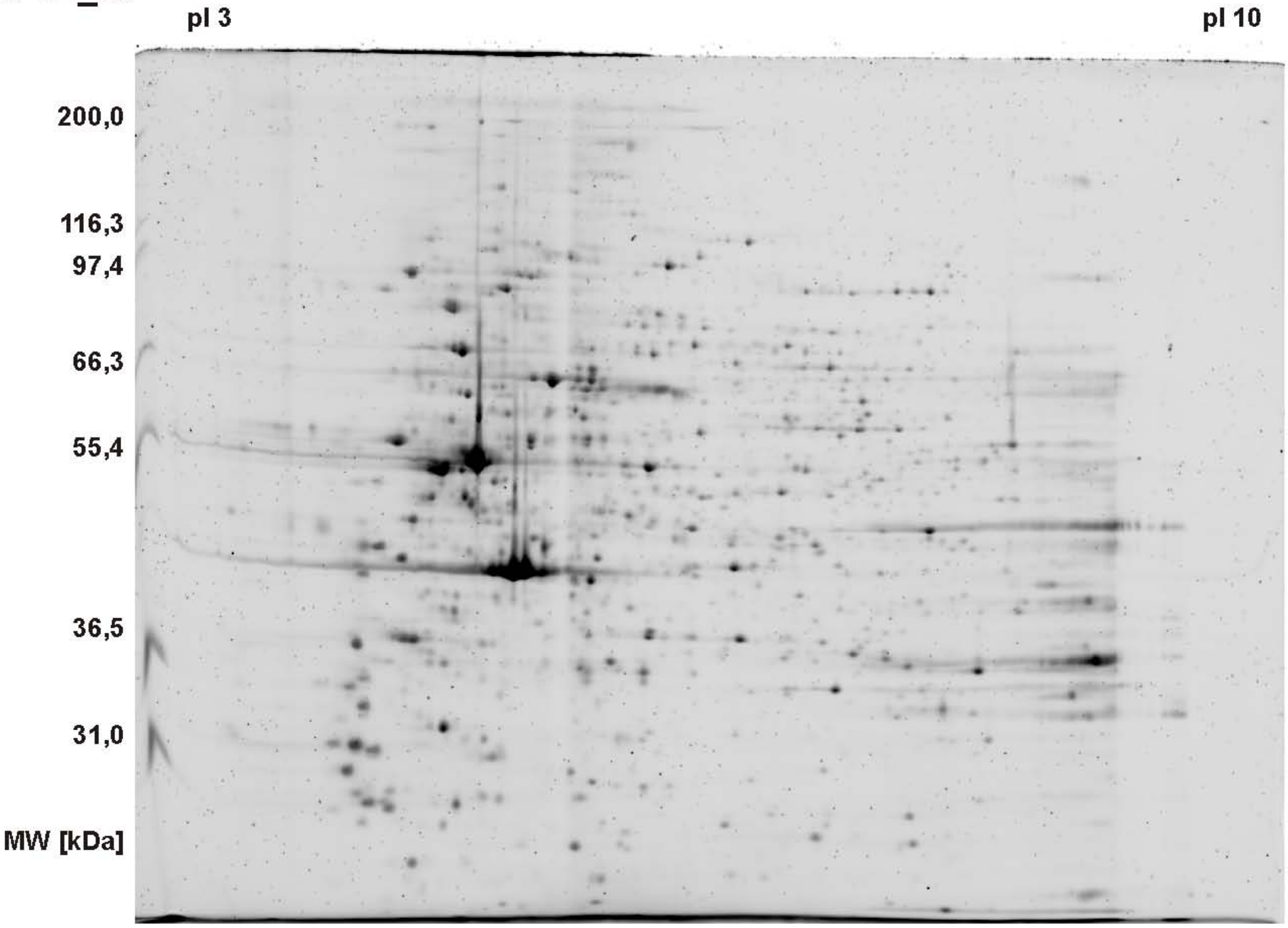

BJ-TER_01

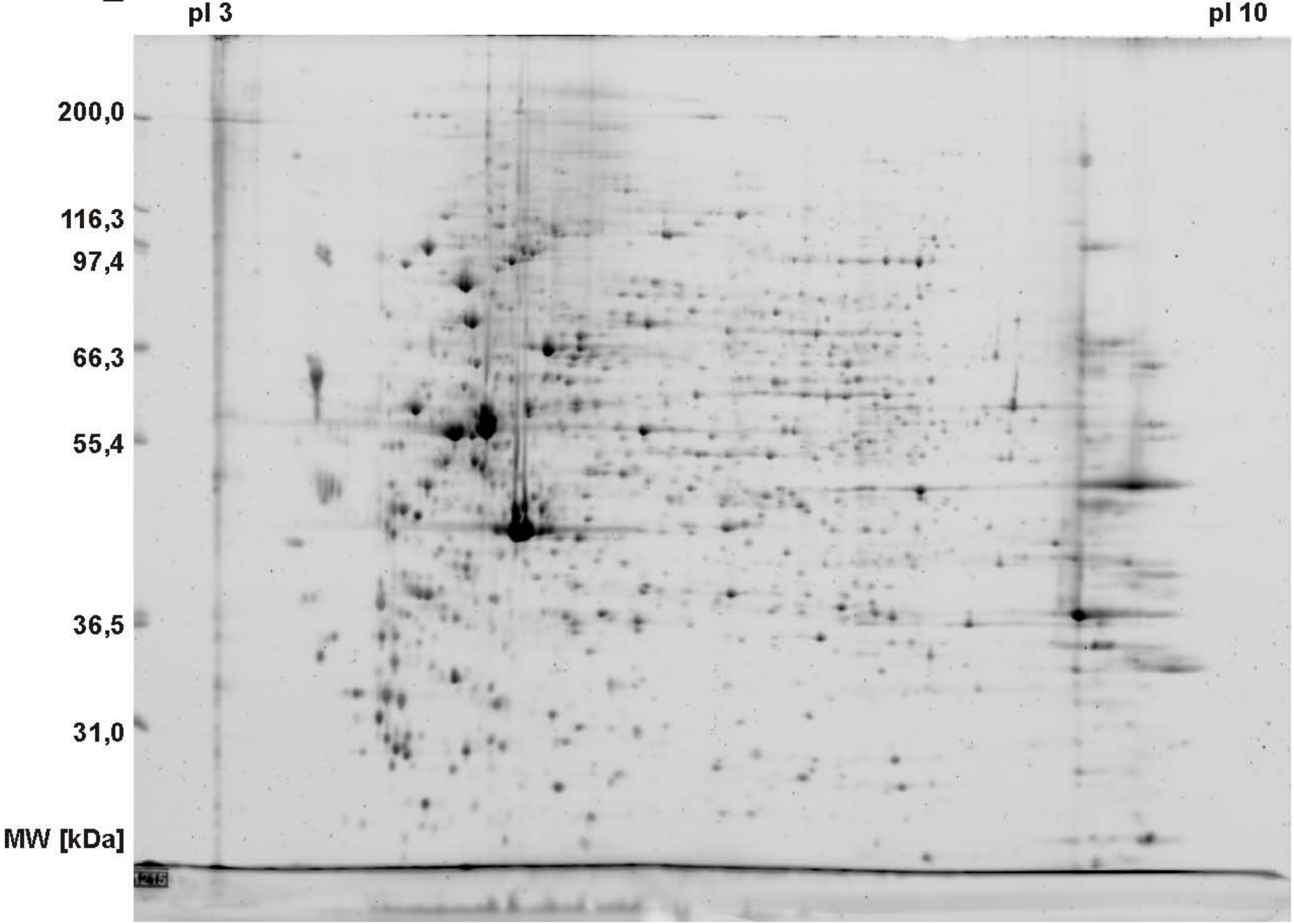

BJ-TER_02

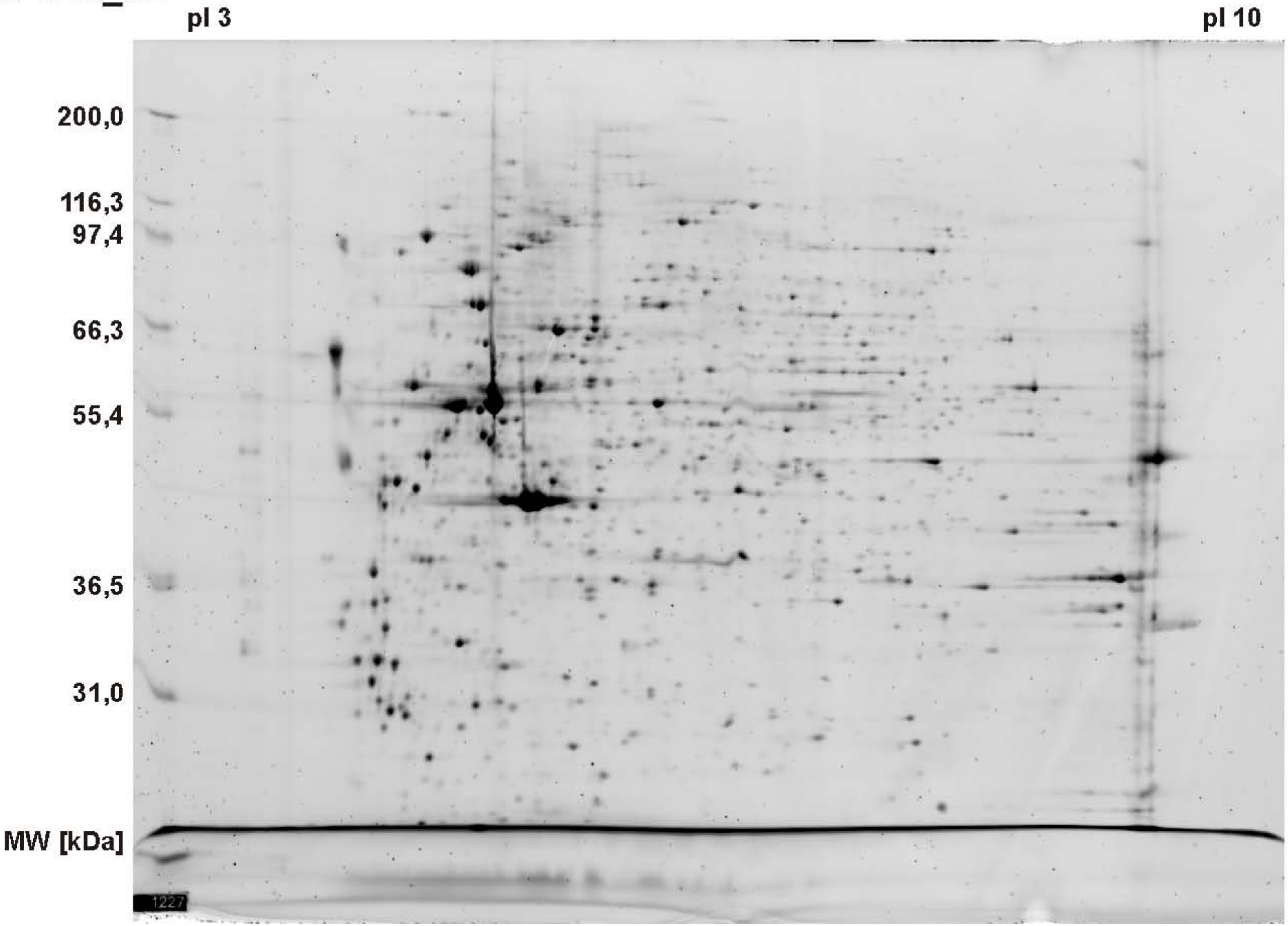

BJ-TER_03

pI 3 pI 10

200,0
116,3
97,4
66,3
55,4
36,5
31,0
MW [kDa]

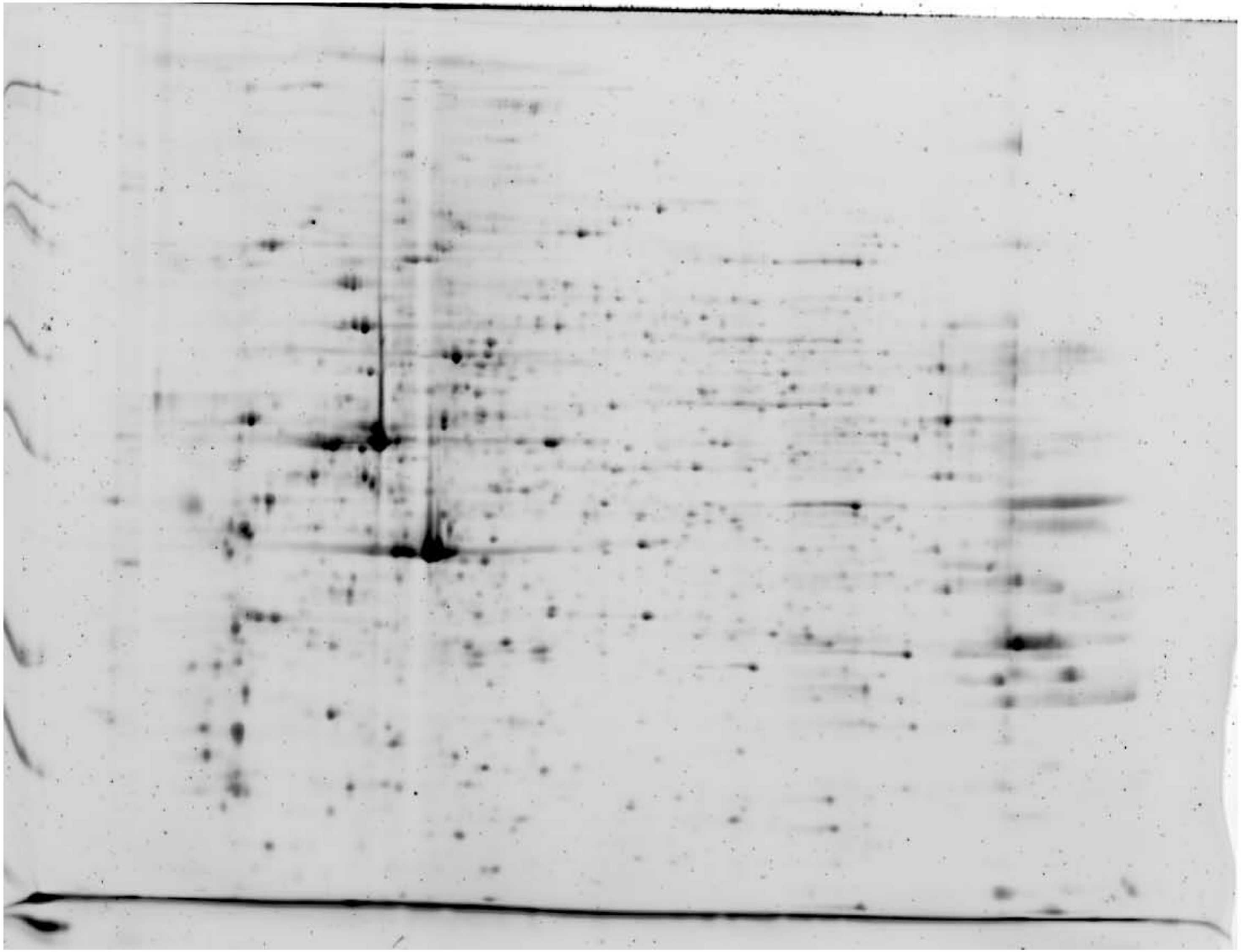

BJ-TER_04

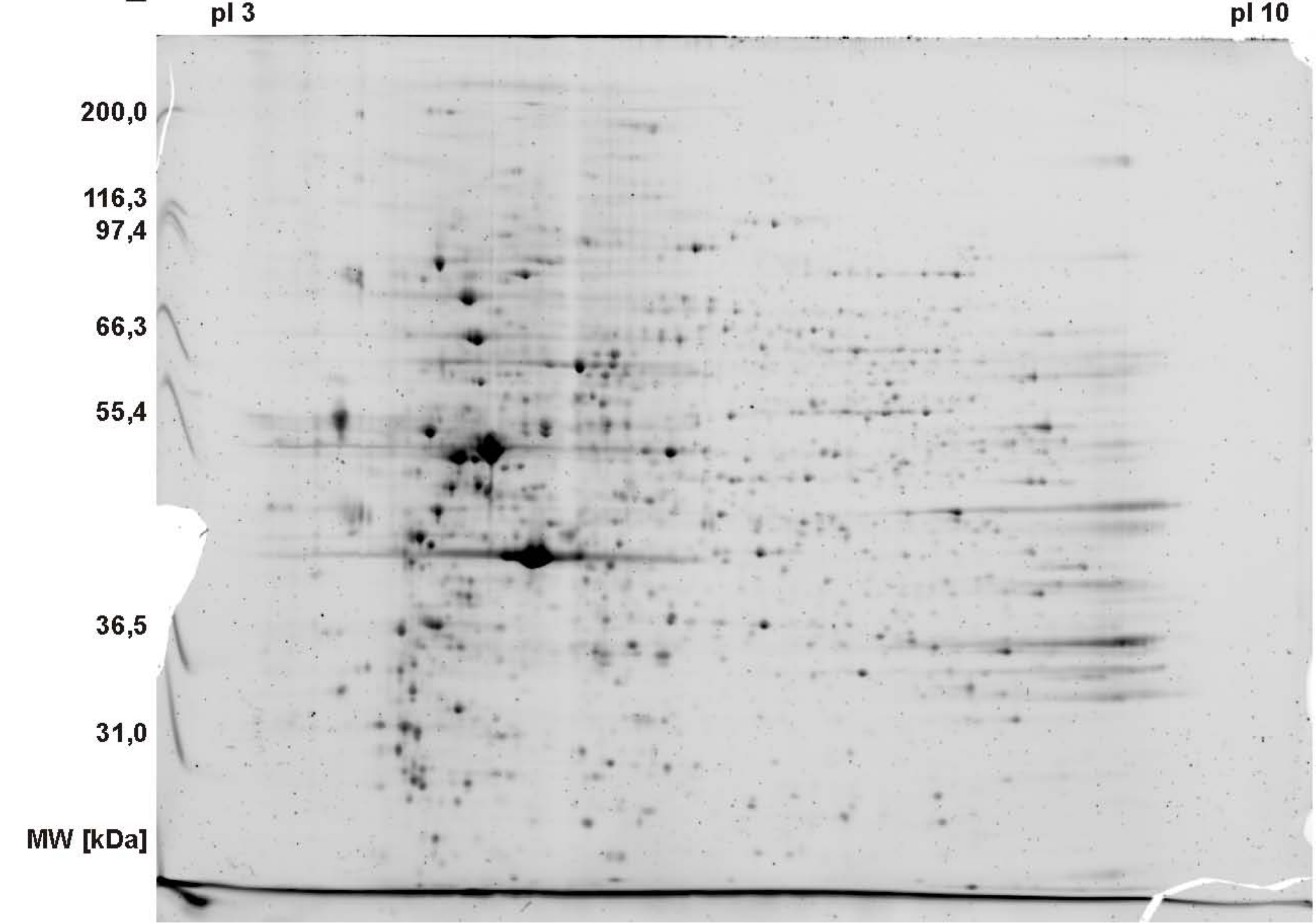

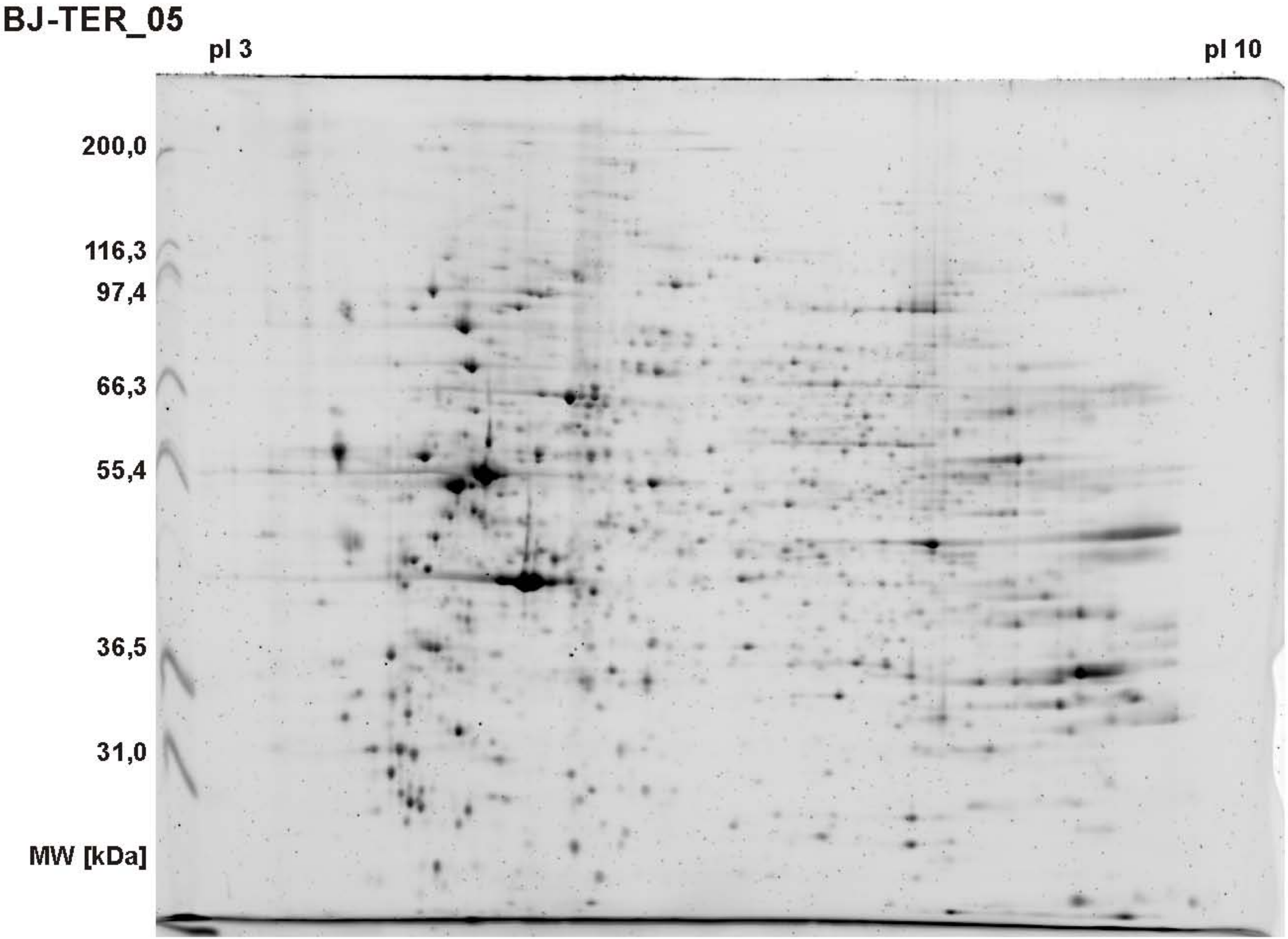
BJ-TER_05
pI 3
pI 10
200,0
116,3
97,4
66,3
55,4
36,5
31,0
MW [kDa]
